# The red bayberry genome and genetic basis of sex determination

**DOI:** 10.1111/pbi.12985

**Published:** 2018-08-10

**Authors:** Hui‐Min Jia, Hui‐Juan Jia, Qing‐Le Cai, Yan Wang, Hai‐Bo Zhao, Wei‐Fei Yang, Guo‐Yun Wang, Ying‐Hui Li, Dong‐Liang Zhan, Yu‐Tong Shen, Qing‐Feng Niu, Le Chang, Jie Qiu, Lan Zhao, Han‐Bing Xie, Wan‐Yi Fu, Jing Jin, Xiong‐Wei Li, Yun Jiao, Chao‐Chao Zhou, Ting Tu, Chun‐Yan Chai, Jin‐Long Gao, Long‐Jiang Fan, Eric van de Weg, Jun‐Yi Wang, Zhong‐Shan Gao

**Affiliations:** ^1^ Institute of Fruit Science College of Agriculture and Biotechnology Zhejiang University Hangzhou China; ^2^ Hangzhou 1 Gene Ltd Hangzhou China; ^3^ Forestry Technology Extension Center Yuyao Ningbo China; ^4^ The National Key Facility for Crop Gene Resources and Genetic Improvement (NFCRI) Institute of Crop Science Chinese Academy of Agricultural Sciences Beijing China; ^5^ Shanghai Center for Plant Stress Biology, and National Key Laboratory of Plant Molecular Genetics Center of Excellence in Molecular Plant Sciences Chinese Academy of Sciences Shanghai China; ^6^ Institute of Crop Science & Institute of Bioinformatics College of Agriculture and Biotechnology Zhejiang University Hangzhou China; ^7^ Forest & Fruit Tree Institute Shanghai Academy of Agricultural Sciences Shanghai China; ^8^ Institute of Forestry Ningbo Academy of Agricultural Science Ningbo China; ^9^ Shunmei Breeding and Propagation Centre for Chinese Bayberry Yuyao China; ^10^ Forestry Technology Extension Center Cixi China; ^11^ Plant Breeding‐Wageningen University and Research Wageningen The Netherlands; ^12^ Present address: Annoroad Gene Tech. Co., Ltd Beijing China

**Keywords:** *Morella rubra*, genome, sex‐determining region, sex‐linked marker

## Abstract

*Morella rubra*, red bayberry, is an economically important fruit tree in south China. Here, we assembled the first high‐quality genome for both a female and a male individual of red bayberry. The genome size was 313‐Mb, and 90% sequences were assembled into eight pseudo chromosome molecules, with 32 493 predicted genes. By whole‐genome comparison between the female and male and association analysis with sequences of bulked and individual DNA samples from female and male, a 59‐Kb region determining female was identified and located on distal end of pseudochromosome 8, which contains abundant transposable element and seven putative genes, four of them are related to sex floral development. This 59‐Kb female‐specific region was likely to be derived from duplication and rearrangement of paralogous genes and retained non‐recombinant in the female‐specific region. Sex‐specific molecular markers developed from candidate genes co‐segregated with sex in a genetically diverse female and male germplasm. We propose sex determination follow the ZW model of female heterogamety. The genome sequence of red bayberry provides a valuable resource for plant sex chromosome evolution and also provides important insights for molecular biology, genetics and modern breeding in Myricaceae family.

## Introduction

**Figure 1:**
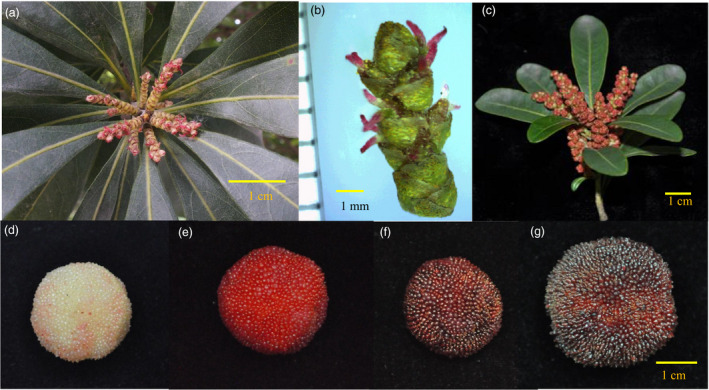
Red bayberry flower and fruit. a and b, female flower. c, male flowers. d to g, the fruits of red bayberry cultivars Y2012‐145, Xiazhihong, Biqi and Dongkui respectively.

Red bayberry (*Morella rubra* or *Myrica rubra*) is an evergreen fruit tree native to China (Jia *et al*., 2015), and has been introduced to Japan, Australia and the United States. It is the only edible fruit species in the family Myricaceae, order Fagles, which is cultivated (Chen *et al*., 2004). The Myricaceae family has three genera, *Morella* is the largest genus with about 50 species distributed in warm, humid regions across Asia, Europe, Africa and the Americas (Huguet *et al*., 2005; Wilbur, 1994). The species in this family are dioecious in general with a very few monecious individuals (Wilbur, 1994). The Chinese name for *M. rubra* is ‘Yang Mei’, in reference to its dioecy characteristic resembling Yang (*Populus*), whereas its drupe fruit type and taste is similar to Mei (*Prunus mume*). Red bayberry has become an important economical fresh fruit in China with an annual production of 1.5 million tons, and processed fruit juice has also entered the USA and European markets. Its fruit was palatable with a special terpene aroma (Cheng *et al*., 2015), and is valued for its remarkably high content of anthocyanins, vitamin C and antioxidant compounds (Bao *et al*., 2005; Zhang *et al*., 2015b). The dioecious male and female flowers, and different fruit colours are shown in Figure 1. It is diploid (2*n* = 2*x* = 16) (Sugiura, 1927) with a small genome size of 323 Mb (Jiao *et al*., 2012). Despite having a very long history of human utilization, breeding programs have only recently been established, with the main challenge in red bayberry breeding being the selection of female individuals with extended fruit shelf life.

In general, sex determination of dioecy, with separate female and male individuals, is controlled by a heteromorphic sex chromosome or a sex determining region on homomorphic chromosomes (Charlesworth, 2013). Genetic and genomic research in a limited number of plants has revealed the prevalent system where the sex of an individual is determined by a pair of sex chromosomes, with females homozygous XX and males heterozygous XY (male heterogamety) (Ming *et al*., 2011) such as in papaya (Wang *et al*., 2012), diploid persimmon (Akagi *et al*., 2014) and kiwifruit (Akagi *et al*., 2018). A few plant species have a ZW system, where the sex of an individual is determined by the genotype of the egg‐cell, and males are the homogametic ZZ and female the heterogametic ZW (female heterogamety), as in poplar (Yin *et al*., 2008), willow (Pucholt *et al*., 2015, 2017) and a wild species of allo‐octoploid strawberry (Tennessen *et al*., 2016; Wei *et al*., 2017). The system for sex determination in red bayberry, which is critical for modern breeding, has been unclear until now.

Currently, no completed whole‐genome sequences have been reported in the Myricaceae family and, in the order Fagales, only the monoecious birch (Salojarvi *et al*., 2017; Wang *et al*., 2013) and walnut (Martinez‐Garcia *et al*., 2016) have been sequenced. Here we report a high‐quality genome assembly of a diploid female and male red bayberry (2*n* = 16 chromosomes) and their genome annotation, the first genetic linkage map in *M. rubra*, characterization of the mechanism controlling sex determination.

## Results

### Genome assembly and annotation

The red bayberry female elite breeding line ‘Y2012‐145’ and a male individual (H2011‐12) were used for genome sequencing (basic plant characteristics shown in Figure S1 in the Supporting Information). Analysis of the 17‐mer sequence revealed the heterozygosity of female was 0.56% and male 0.70% (Figure S2). For Y2012‐145, a total of ~278‐fold coverage of Illumina paired‐end reads were first assembled by SOAP*denovo2* (Luo *et al*., 2012) (V2.04.4), and ~15‐fold coverage of PacBio data assembled by Falcon (Chin *et al*., 2016) (v1.7.4) (Table S1), were merged using the HABOT software (Zou *et al*., 2017). The final assembly of the female genome was 313 Mb (NCBI Sample Project NO. SAMN07510764) covering 96.9% of the bayberry genome (323 Mb). A total of 1114 scaffolds and 3433 contigs were assembled with an N50 of 635 kb and 193 kb respectively (Table S2). The assembled male individual H2011‐12 genome was 313 Mb using 48‐fold Illumina reads and 61‐fold coverage by Pacbio reads (Table S1), the same size as the female, accounting for 98% of the total of 319 Mb, and the N50 contig index was 1.1 Mb (Table S2).

A bi‐parental genetic linkage map was constructed by JoinMap^®^ 4.1 using an F_1_ population (Biqi × Dongkui) of 95 individuals with SNP markers identified by RAD‐Seq and alignment to scaffold and contig sequences (Table S3). The final map spanned 531‐cM across eight linkage groups and contained 3075 SNP markers aggregated to haplotype blocks (HB) representing 407 genetic bins (Figure S3 and Table S4). The genome‐wide heat map for recombination frequencies for all possible haplo‐block marker pairs is presented and the centromere region for each chromosome was determined (Figure S4 and Table S4 **)**. Sequence alignment by Blast with primary assembled contigs from both the male and female genome, and the order of SNP markers in the linkage map, helped connect and orientate contigs and scaffolds. This integrated approach greatly contributed to the quality of the reference female and male red bayberry genome, with the N50 scaffold parameter increased from 0.6 Mb to 1.6 Mb for the female and from 1.1 Mb to 2.0 Mb for the male (Table 1 and Table S2). As part of this process, the initial 169 female scaffolds and 12 male scaffolds were split, and a total of 531 scaffolds were anchored to the eight pseudochromosomes for the female, comprising 90% (280‐Mb) of the Y2012‐145 genome assembly, 87% coverage of the whole genome (Figures S5 and S6a), enabling orientation of 228 of the anchored scaffolds (267‐Mb, 95% of the total anchored sequences). The H2012‐12 male genome was also assembled into eight pseudo‐chromosomes, 264 Mb in length, comprising 84% of the total assembly (313 Mb) (Figure S6b). Comparison of the genetic and physical distances between SNP markers revealed a single region of distinct recombination suppression on each of the eight pseudo‐chromosomes, indicating the centromere regions of the chromosome of about 2 cM each that were represented by 3–4 haploblocks (Figures S3, S5 and Table S4). Some short scaffolds in the centromere regions could not be fine mapped due to lack of recombination and lack of overlapping synteny between the female and male.

**Table 1:** Characteristics of the *M. rubra* (2*n* = 16) genome assembly and annotation

Categories	Female	Male
Estimate of genome size (by *k*‐mer)	322.7 Mb	319.2 Mb
Total size of assembled scaffold	312.6 Mb	313.5 Mb
Number of scaffold (≥10 Kb)	859	1407
N50 (scaffold)	1.6 Mb	2.0 Mb
Heterozygosity	0.56%	0.72%
Number of anchored scaffold	275	301
Anchored scaffold size	280 Mb	264 Mb
Number of gene models	29 414	26 416
Total size of TEs	115.0 Mb	154.6 Mb
TE share in genome	36.7%	49.3%

We assessed the quality of the assembly by four independent methods. For the female genome, we first randomly selected ten scaffolds longer than 30 Kb and used the WGS paired‐end reads to check the assembly accuracy within scaffolds. The results showed all regions can be covered by paired‐ends reads (Figure S7). Second, 94.5% of a total of 61,391 transcriptions derived from the *de novo* assembled RNA‐seq data of five tissues of Y2012‐145 were successfully aligned to the assembled genome, and 94.7% of the fruit EST sequences from *M. rubra cv*. Biqi (Feng *et al*., 2012) were retrieved (Table S5). Third, the completeness of the assembly was evaluated using the BUSCO (Simao *et al*., 2015) (Benchmarking Universal Single‐Copy Orthologs) data sets, with about 94% of the core eukaryotic genes retrieved (Table S6). Finally, the positions and orientation of assembled contigs from both the female and male genome generally had good linear synteny at a sequence identity threshold of 85% (Figure S8) and correlation of their relative positions on the reference genetic linkage map. For the male genome, 96% of a total of 99 328 transcriptions derived from the *de novo* assembled RNA‐seq data of male buds and flowers of H2011‐12 were successfully aligned to the assembled genome (Table S5).

Genome annotation was primarily done in the female Y2012‐145, with complementary additional genes annotated in the male genome. The female genome had 114 Mb repetitive sequences, accounting for 36.4% of the assembly (313 Mb), whereas the male had 154.6 Mb repetitive sequences, accounting for 49.3%, which is more reliable because of higher coverage of long PacBio reads. Based on the known repeat motifs, 82.9% of the repeat sequence were classified and annotated. The retrotransposons (class I elements) constituted 21.4% of the genome, with superfamily *gypsy* and *copia* retrotransposons (9.9% and 5.1% respectively) (Table S7 and Figure S9), showing high similarity to that in peach (Verde *et al*., 2013). The repeat divergence rate peaked at 30%, and more than 98.8% of TEs had a divergence rate of >10%, indicating that most red bayberry TEs are of relatively ancient origin, similar to jujube (Liu *et al*., 2014) and mulberry (He *et al*., 2013) (Figure S10).

To annotate the female bayberry genome for protein‐coding genes, we used a combination of *ab initio* gene predictions and homologs sequence searching, integrated with RNA‐seq data from different tissues (Table S8). We predicted 29 414 protein‐encoding genes with an average coding sequence length of 1144 bp and five exons per gene (Table S9). Additionally, 26 460 genes in the male genome were predicted and supported by transcripts from the male flowers (Table 1), of which 3079 genes were not covered by the female gene set, so the total gene number was 32 493 for both female and male. The number of genes was similar to that found in peach (Verde *et al*., 2013) and pomegranate (Yuan *et al*., 2018), but considerably lower than that in apple (Daccord *et al*., 2017). A total of 26 316 (89.5%) genes in the female had substantial homology with those in public databases (Table S10). In addition to protein‐coding genes, we identified 1158 putative transcription factor genes distributed across 58 families (Table S11), 489 rRNAs, 205 snRNAs, 626 tRNAs and 128 miRNAs (Table S12). Tissue‐specific expressed genes based on the RNA‐seq data were analysed (Figure S11). Most of the root‐specific genes appear to be involved in plant defence and stress responses, such as plant disease resistance genes. A total of 26 152 genes were mapped on the eight female pseudo‐chromosomes. Self‐alignment of the bayberry genome sequences based on the 26 152 gene models identified 6851 paralogous gene groups and 893 gene blocks, indicating that the bayberry genome may have undergone frequent inter‐chromosome fusions and segmental duplication during its evolutionary history (Figure S12).

### Genome evolution analysis

**Figure 2:**
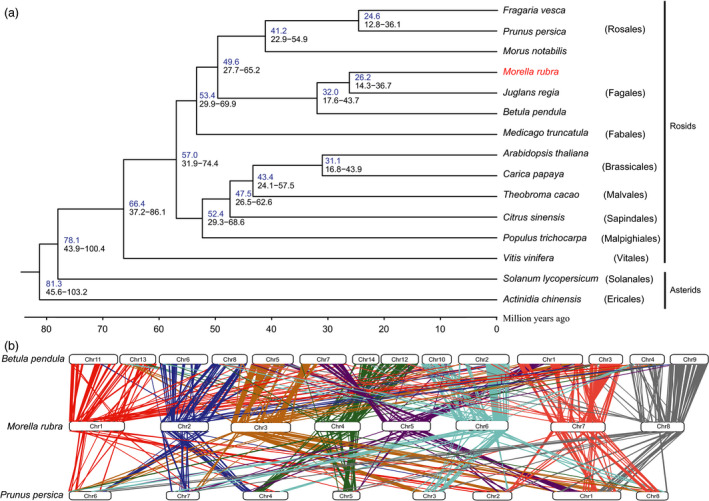
Genome evolution and comparative analysis in the red bayberry genome. (a) Phylogenetic analysis of red bayberry and other sequenced plants. *Actinidia chinensis* was used as outgroup. (b) Syntenies among red bayberry (pseudochromosomes 1–8), peach (chromosome 1–8) and silver birch (chromosome 1–14).

A phylogenetic tree based on single‐copy genes of red bayberry together with 12 other sequenced Rosids genomes, two Asterids genomes, and *Actinidia chinensis* as outgroup (Figure 2a and Table S13). The result showed *M. rubra* to be relatively close to *Juglans regia* and *Betula pendula*, as they belong to the Fagales order, and indicate a divergence time of 26.2 Mya for *M. rubra* and *J. regia*. Furthermore, our results support Fagales being close to Rosales and Fabales, which is in accordance with the classification of flowering plants (Allantospermum *et al*., 2016).

The 4DTv (fourfold synonymous third‐codon transversion) value peaked around only 0.50 in red bayberry, as expected for a species with an ancient triplication common to eudicots (Tang *et al*., 2008a) while lacking recent whole‐genome duplication (Figure S13). The orthology between *M. rubra* and *J. regia* or *M. rubra* and *B. pendula* showed 4DTv distance peaks at ~0.15 and ~0.16, respectively, indicating the divergence time between *M. rubra* and *B. pendula* was earlier, consistent with the phylogenetic tree (Figure 2a). All 4DTv values among paralogs in *M. rubra*,* B. pendula*,* J. regia*,* M. truncatula*,* M. notabilis* and *P. persica* peaked at ~0.50, indicating that the hexaploidy in these species occurred at a similar time and before the split of Fagales, Fabales and Rosales.

We went on to compare syntenic relationships in red bayberry, peach and silver birch. A total of 462 (red bayberry vs silver birch) and 247 (red bayberry vs peach) orthologous gene pair blocks were found and used to visualize detailed orthologous chromosome‐to‐chromosome relationships (Figure 2b). The complicated syntenic patterns indicated a high level of chromosomal rearrangements between red bayberry and peach, while each red bayberry chromosome mainly matched two birch chromosomes, possibly from the ancient gamma hexaploidy event (Salojarvi *et al*., 2017).

Red bayberry genes, identified by comparative analyses with those in mulberry, papaya, peach and poplar, clustered in 20 015 orthologous gene groups. A large number of shared genes were found in most plant species. A total of 8432 were shared among all five genomes, 1083 were confined to Fabidae (red bayberry, mulberry, peach and popular), 260 to plants with fleshy fruits (red bayberry, mulberry, peach and papaya) and 1330 were unique to *M. rubra* (Figure S14). Further functional characterization revealed that fruit‐specific gene families were highly enriched in biosynthesis of secondary metabolites. About 45.4% of the red bayberry‐specific genes could be annotated as ‘transferase activity’ and ‘membrane’ in GO terms (Table S14).

### Identification of a sex‐specific genomic region

**Figure 3:**
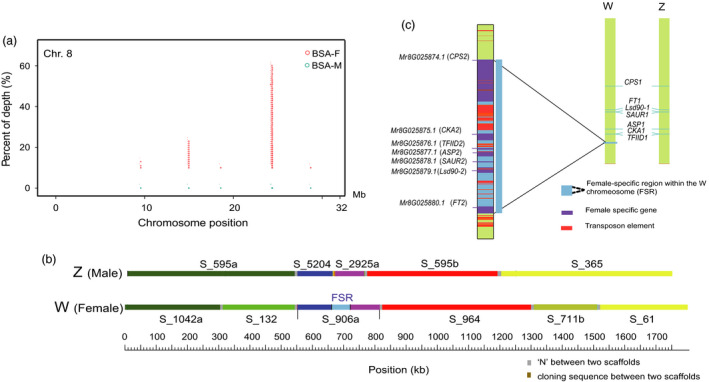
Identification of female‐specific region (FSR) and sex‐determination ZW model in red bayberry. (a) A female‐specific genomic region was found on chromosome 8 based on BSA reads from male and female samples (BSA‐M and BSA‐F respectively). (b) Schematic alignment of the boundary scaffolds from both W (based on female) and Z (based on male) chromosome around the FSR by BLASTN, a gap of 1.4 kb was filled between S_5204 and S_2925a by nested PCR amplification. (c) ZW model of chromosome 8 and the candidate genes in the female‐specific region.

To analyse sex determination in red bayberry, bulked DNA pools of female (BSA‐F, including 100 varieties) and male (BSA‐M, including 100 natural individuals) DNA from *M. rubra* were used to construct libraries (Table S15), and sequenced with 106‐fold and 127‐fold coverage respectively. Three female cultivars and three male individuals were also resequenced individually with an average 44‐fold coverage (Table S16). The BSA‐F and BSA‐M reads were aligned to both the female and male reference genome to detect true or ghost (Ghost Ins/Del result from paralogous regions with small sequence divergence to the reference) SNPs and Ins/Dels associated with the male and female. For Scaffold S_906a (262‐kb) anchored to chromosome 8 in HB8‐35 (Figures S3 and S15), we found the S_906a‐140,699 SNP to be differently represented, being heterozygous in the BSA‐F (A:G signal intensity was ~1:2 ratio, with A the female reference), while male BSA were almost homogenous for G (97%): this pattern was confirmed with seven resequenced female and males. This difference in BSA‐F and BSA‐M sequences was repeated in other candidate SNPs flanking S_906a on chromosome 8, but did not fit all resequenced individuals (Table S17). In addition, abundant ‘Insertions’ of more than 100 consecutive base pairs were found in BSA‐F with significantly higher (66% of 106‐fold) sequence coverage found in chromosome 8 (Figures 3a and S16), located in Scaffold_906a (Figure 3b). However, using the male genome as template, we found no outstanding SNPs or insertions from the BSA‐M associated with sex differentiation, although there were many minor contrasts across each pseudochromosome at a relatively low signal intensity (less than 40% coverage of 127‐fold total read depth) in the male bulked sample (Figure S17). These results indicate sex in *M. rubra* is determined by a single genomic region in the female genome. According to the linkage mapping and assembled contigs/scaffold alignments of from both female and male, we confined the female‐specific region (FSR) in the middle of S_906a to a 59‐kb stretch (Figure 3b) that included seven putative genes *MR8G025874.1*‐*MR8G025880.1* (Figure 3c and Table S18). The 1.4 kb gap with repeat sequences between S_5204 and S_2925a was filled by PCR amplification with primers designed from two ends of adjacent scaffolds (Figure 3b). Sequence analysis of the FSR revealed highly repetitive transposon sequences, such as *hAT*, MULE and LTR‐*copia* in the middle and two ends (Figure 3c), similar to sex‐specific regions in other species, such as persimmons and papaya (Akagi *et al*., 2014; Bachtrog, 2013; Wang *et al*., 2012).

### Characterization putative genes in a sex‐specific genomic region

**Figure 4:**
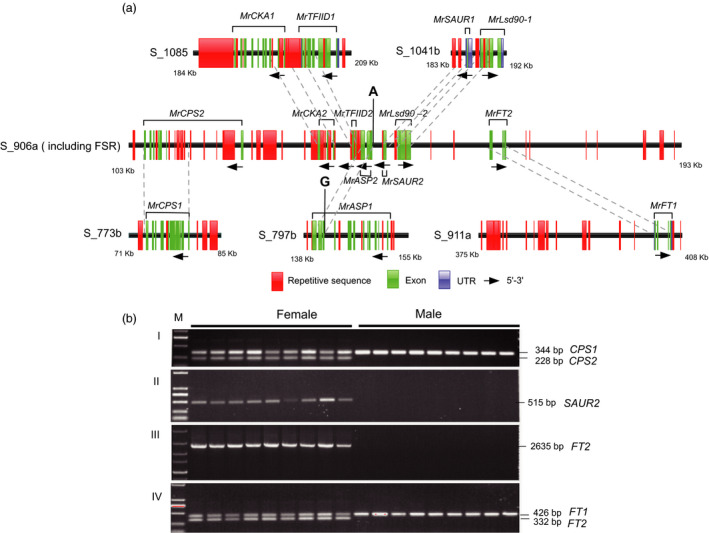
Genomic structure comparison between the putative genes in FSR and corresponding paralogous genes leading to development of sex‐linked markers. (a) The schematic gene structures and repetitive sequence in FSR and their paralogous. The red boxes are repetitive sequence around the genes, green boxes are exons, the blue box a UTR region, dash lines link similar regions of the female‐specific gene and paralogous present in different scaffolds. (b) Sex‐linked markers derived from three candidate genes. I and IV, co‐dominant sex‐linked markers derived from *MrCPS2* and *MrFT2* in female and male *M. rubra*. II and III dominant female‐specific markers from *MrSAUR2* and *MrFT2*.

By blasting the protein sequence of seven genes in the female‐specific region against the TAIR database, we found three genes related to flowering: *MR8G025875.1* (*MrCKA2*) encodes the casein kinase II (CK2) regulating flowering time (Ogiso *et al*., 2010); *MR8G025877.1* (*MrASP2*) is SUMO protease 1, positively regulating the transition to flowering (Kong *et al*., 2017); and *MR8G025880.1* (*MrFT2*) predicated as the *FLOWERING LOCUS T* (*FT*) gene, assumed to be a major component of florigen, that regulates flowering in *Arabidopsis* (Fornara *et al*., 2010). Two genes are related to hormones. *MR8G025874.1* (*MrCPS2*) has been predicated as an ent‐copalyl diphosphate synthase (*CPS*), a key enzyme in the biosynthesis of gibberellin (GA) (Hedden and Phillips, 2000), and *MR8G025878.1* (*MrSAUR2*) as a member of a SAUR‐like auxin‐responsive protein family (SAUR58) (Wuest *et al*., 2010), which induce early auxin‐responsive genes. *MR8G025876.1* (*MrTFIID2*) predicated as transcription initiation factor TFIID. *MR8G025879.1* (*MrLSD90‐2*) was annotated as a gene involved in the metabolism of very long‐chain fatty acid‐containing phospholipids (Yokoyama *et al*., 2008). Also on chromosome 8, for each candidate gene in the FSR one copy of a paralogous gene was present at different loci (Figures 3c, 4a and Table S18). To confirm the coding sequence of these putative genes, they were sequenced after amplification with gene‐specific primers on the cDNA templates from female buds. Only the first predicated exon were obtained for *MrCPS2* and *MrFT2*, and full length sequences were verified for *MrCKA2*,* MrTFIID2*,* MrASP2*,* MrSAUR2* and *MrLsd90‐2*. The specific female S_906a‐140,699A SNP mentioned above was located 246 nt before *MR8G025877.1* (*MrASP2*). S_906a‐140,699G SNP is actually from a paralogous gene *MR8G020746.1* (Figure 4a). Interestingly, comparative analysis of the FSR and their paralogous sequences shows *CPS2*,* CKA2*,* ASP2*,* SAUR2*,* Lsd90‐2* are partially matched to their paralogous and abundant repetitive sequences among the FSR and their paralogous sequences (Figure 4a), suggesting that the evolution of the bayberry FSR region may come from the trans‐ and cis‐ duplication.

The next stage was to develop sex predictive DNA markers. A single amplified band was detected only in the female individuals (including two parents of our mapping population) with markers based on *Mr8G025878.1* (*SAUR2*) and *Mr8G025880.1* (*FT2*) (Figure 4b). The presence and absence of these two markers in the mapping population was close to 3 : 1 ratio as expected. We also designed sequence characterized markers for each gene pair *MrFT1*/*MrFT2* and *MrCPS1*/*MrCPS2* based on sequence polymorphism in introns (Figure S18). The female‐specific fragments were amplified along with the amplicon of the second locus (paralogous genes present in both male and female) as a positive internal control. The two co‐dominant markers (Table S19) amplified two bands in the female, but one in the male (Figure 4b), and those three markers for *MrCPS2*,* MrFT2* and *MrSUR2* were perfectly valid in tests with 133 female and 128 male individuals (Table S15) collected from different geographical sources in China. Only the female‐specific band segregated in the mapping population. Further characterized sex‐linked S_906a_MrFT2_BD markers specific to each parent (both are female in normal status) of the mapping population ‘Biqi’ × ‘Dongkui’ (Figure S15) were vital to map this gene, and S‐906a on LG8, which helped to split the initial scaffold S_906 in two, one is S‐906a (contig 1 and contig 2, 262 Kb) containing the sex‐specific region on LG8 and the other S‐906b (contig 3 and contig 4) on LG2. The female‐specific region (FSR) was located in haploblock (HB8‐35) of about 500 kb, with boundary contigs/scaffolds aligned to contigs from the male (Figures 3b and S15), suggesting that the FSR region in *Morella* might be a small region flanked by recombinant regions. Comparing the female and male assembled sequences and perfect link of the molecular markers to sex, we put forward the hypothesis of a female heterozygous ZW sex‐determining model in red bayberry: females have a genome region (W) lacking in males, which have the ZZ genotype.

### Female and male flower development and expression of sex candidate genes

**Figure 5:**
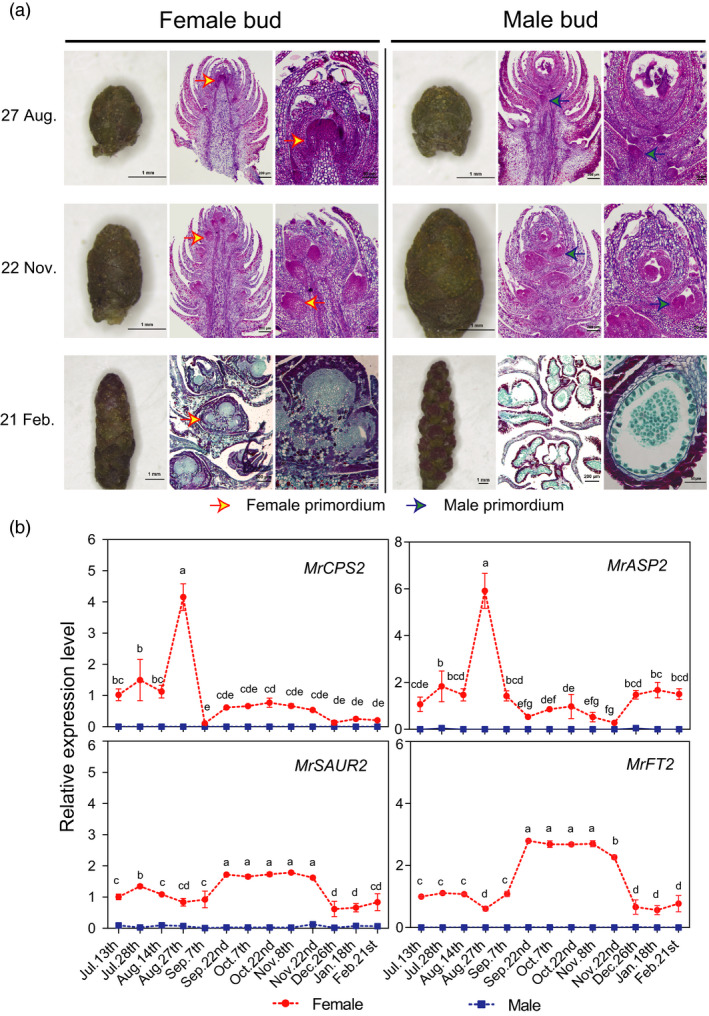
Female and male floral bud development and expression profiles of four female‐specific candidate genes. (a) Morphology and cross‐sectional images of female and male flower initiation and development from July to February. (**b**) Quantitative expression level of four female‐specific candidate genes: *MR8G025874.1* (*MrCPS2*), ent‐copalyl diphosphate synthase. *MR8G025877.1* (*MrASP2*) is SUMO protease; *MR8G025878.1* (*MrSAUR2*), SAUR‐like auxin‐responsive protein family; *MR8G025880.1* (*MrFT2*) predicated as *FLOWERING LOCUS T* (*FT*) gene.

After fruit harvest in June, the vegetative bud transits to flower buds from July to August, the pistil and stamen primordium form between September and November, and the flower type is visible in February (Figure 5a). The female primordium appears first on the upper part of the bud, whereas the male primordium appears first in the lower part. The expression patterns of four candidate genes in FSR together were investigated. Clearly, four female‐specific genes were expressed only in the female bud, *MrCPS2* and *MrASP2* expression reached a peak on August 27 at the female flower initiating stage, followed by higher *MrSAUR2,* and *MrFT2* expression during the flower primordium formation period (September to November, Figure 5b). This study of the serial anatomy of flower and candidate gene expression study demonstrated the roles of two candidate genes, *ASP* and *CPS*, as key initiating factors.

## Discussion

The red bayberry genome size is small and simple (around 320 Mb), being diploid with intermediate heterozygosity. The female and male independent assemblies, reciprocal sequence comparison at micro level in contigs and an efficient mapping approach (Di Pierro *et al*., 2016), led to high quality of assembly, with an extended total and oriented size of the female pseudochromosome of 280 Mb (87%) and 264 Mb (82%) of the male (Table 1). Due to genetic diversity of two parents, 65% of haplotype blocks (HB) in the linkage map was heterozygous (Figure S3), which is helpful to define HB and anchor scaffolds in right position and orientation.

Myricaceae, a member of the Fagales order according to Angiosperm Phylogeny Group IV, is closely related to Cucurbitales, Rosales and Fabales (Allantospermum *et al*., 2016). Our results support this classification. As one of IV clades of actinorhizal plants, the genetic and fossil record puts Myricaceae close to Betulaceae and Casuarinaceae (Huguet *et al*., 2005) (the member of Fabales). The time and centre of origin of Myricaceae has been proposed by two distinct hypothesis: late Cretaceaous (−90 Mya) in South eastern Asia (Chourey, 1974) or Early Tertiary (−46 Mya) in north America (Huguet *et al*., 2005). Our evolution analysis indicates that red bayberry occurred about 49 Mya ago (Figure 2a), thus supporting the latter hypothesis of a recent event close to Early Tertiary.

*Morella rubra* is dioecious fruit tree. Dioecious plants only account for 6% in the flowering plant (Charlesworth, 2002; Ming *et al*., 2011). The diversity of genetic mechanisms for sex determination in these plants is attributed to how the separate sexes evolved independently from hermaphroditism in different lineages, and often recently (Pannell, 2017; Renner, 2014). Genetic polymorphism (sex chromosome) is thought to be the cause of dioecy, with the XY system being prevalent (Ming *et al*., 2011; Pannell, 2017), whereas the ZW system appears in very few genera (Ming *et al*., 2011) such as *Ginkgo*,* Datisca*,* Populus* (Yin *et al*., 2008), *Salix* (Pucholt *et al*., 2015, 2017), *Fragaria* (Tennessen *et al*., 2016; Wei *et al*., 2017). Our study adds the *Morella* genus to this short list, and it will become a reference for other dioecious plants in Fagales. The ZW sex‐determining mode seems to be prevalent among the dioecious plants in Cucurbitales, Rosales and Fagales (this study) within Fabidae (Ming *et al*., 2011).

Because of the common origin of the sex‐determining or regulatory genes and repeated sequences within and surrounding the so‐called SDR (Charlesworth, 2013; Ming *et al*., 2011; Moore *et al*., 2016), it is not easy to find, locate and define the exact boundary lines of male‐ or female‐specific regions. Our integrated approach combined DNA marker haploblock mapping, sequencing both female and male genomes and GWAS using two bulked sex DNA pools, has successfully pinpointed a core female‐specific region of approximately 59 Kb FSR as sex‐determining region (SDR) on the distal end of linkage group 8 in *Morella rubra* (Figures 3c and S5). It is the smallest identified to date, with others ranging from 100 Kb to 10 Mb: *Populus* (100 Kb) (Geraldes *et al*., 2015), *Vitis* (143 Kb) (Fechter *et al*., 2012), *Fragaria* (280 Kb) (Tennessen *et al*., 2016), *Salix* (804 Kb) (Pucholt *et al*., 2017) and much larger SDR (1–10 Mb) in *Actinidia* (Zhang *et al*., 2015a) and *Carica* (Wang *et al*., 2012). This 59‐Kb region as a whole was absent in Z chromosome 8 (Figure 3b and c). The origin of this region is likely due to duplication and rearrangement of seven predicted genes, considering evidences of identified *hAT* and MULE and LTR‐*copia* transposable elements and paralogous present on the same chromosome. Examination of sex recombination suppression and sex‐linked differences in SNP intensity through our genetic map was not feasible as our mapping population was created by a cross between two female cultivars (see below) and small size of FSR. Alternatively, we tested three markers covering the 59 kb region on the females and males in a large germplasm (Table S15), they were present collectively in the female, indicating no recombination within the 59‐Kb region and recombination is suppressed within this region. Because of the long juvenile period, most seedlings from the mapping population (Biqi × Dongkui) have not yet borne flower, only one individual appears to be female that fit the ZW genotype as defined by markers. To overcome the general heterozygous status on chromosomes 8 controlling sex determination in ordinary female varieties, we will select homozygous WW females (viable) genotype lines and recombinant lines from our ‘Biqi’ × ‘Dongkui’ (WZ × WZ) mapping population for resequencing, to better illustrate the genomic structural difference of the chromosomes containing putative W and Z regions. As the WW genotype is viable, the *M. rubra* sex chromosome may at the stage 2 of chromosome evolution (Ming *et al*., 2011).

The developed genetic markers linked to female and male plants in red bayberry were successful in all tested individuals from diverse genetic backgrounds, demonstrating their general validity. The sex‐linked markers can be used in routine high throughput early selection on seedlings long before fruiting, accelerating the breeding process of this exotic fruit crop. The sex‐linked Mr‐FT2‐M2 marker, transferred to American *Morella cerifera* with minor modifications of the primers, also perfectly linked to the sex phenotypes of 50 randomly selected females and 50 males (Figure S19). On the basis of this, we would expect similar female‐determining regions in the other two genera within the Myricaceae family. Sex markers can be used in the early selection of female plants for ornamental use, eliminating most male plants that may cause pollen allergy (Jacinto *et al*., 1992).

Natural female and male populations were clearly separated in different groups as illustrated in the phylogenetic tree and population structure analysed with 84 nuclear SSR markers (Jia *et al*., 2015). Two markers (ZJU060 and ZJU130), moderately associated with sex subpopulations (Jia *et al*., 2015), can now be traced to chromosome 3 and 7, respectively, not chromosome 8, implying they are not directly linked to the sex‐determining factors, rather to other characters accompanying sex stratification. With aid of whole‐genome sequences, we have anchored 7 SSR markers to chromosome 8 in our previous study (Jia *et al*., 2015), their allele frequencies were biased moderately between the male and female population, indicating some linkage disequilibrium around the FSR region.

This study provides a basis to further identify and characterize sex‐determining or regulatory genes in red bayberry. The female‐specific region harbours a cluster of candidate genes which may be involved in flower development, as indicated by gene expression when the sex is determined during flower development (Figure 5). The expression pattern of *MrCPS2* (though only the first predicted exon was expressed) and *MrASP2*, suggest they have a role in sex differentiation. Another approach is looking at mutations in nature. A monoecious red bayberry individual (WZ genotype) has female flower and abnormal male catkins, the female *cv*. ‘Biqi’ only very rarely has a mutated branch bearing only male flowers. Plant hormones also have a regulatory effect on the flower sex, as seen in *cv*. ‘Dongkui’, where the normal female plant can be reversed to a monoecious tree with both female and male (the source pollen to construct the mapping population ‘Biqi’ × ‘Dongkui’) after applying the vigour control regulator uniconazole in July and August. However, we have not yet found any male trees have a mutation bearing female flower or fruit in nature. These observations indicated some genetic and environmental modifiers affect the phenotype of ZW. More in‐depth research on the candidate genes will provide new insight of gene expression difference and dosage compensation in sex determination and regulation.

In conclusion, we report the *de novo* whole‐genome sequences, the first in the Myricaceae family, for a male and female individual of red bayberry. The genomes are fundamental to molecular biology, genetics and breeding research on *Morella* species. We identified a conserved sex‐determining region, underlying the ZW genetic model, the female has a specific genomic region with putative genes related to sex floral development.

## Materials and methods

### Genome assembly of both female and male individual

The female'Y2012‐145’ (Jia *et al*., 2014) and male ‘H2011‐12’ (Jiao *et al*., 2012) red bayberry were used for genome sequencing. Different library types were generated for sequencing and an overview of the library types for assembly are shown in Table S1. The red bayberry Genome assembly pipeline is shown in Figure S20. Illumina PE reads were assembled into contigs (pb‐contigs) using SOAP*denovo*2 (Luo *et al*., 2012) with default parameters, and PacBio reads were assembled to contigs (sp‐contigs) using Falcon (Chin *et al*., 2016) (v1.7.4). The assemblies were merged together using the HABOT software (Zou *et al*., 2017) followed by a round of scaffolding and gap filling using Illumina PE reads to obtain the final assembly.

### Genetic map and pseudochromosome construction

An integrated (bi‐parental) genetic linkage map of red bayberry was constructed using the F_1_ population from a cross between *M. rubra cv*. ‘Biqi’ (female) and ‘Dongkui’ (usual female, induced as pollen donor) (Jiao *et al*., 2012; Wang *et al*., 2015). Both parents are highly heterozygous and genetically distant (Jia *et al*., 2015), so the F_1_ population could be considered a double pseudo‐testcross. A total of 95 individual and their parents were genotyped with restriction site‐associated DNA sequencing (RAD‐seq, EcoR1 restriction enzyme), the summary statistics are shown in Table S3. The clean reads were mapped onto the assembly genome using Bowtie2 (v2.2.2) (Langmead and Salzberg, 2012) with the following parameters: ‘–end‐to‐end ‐k 1 ‐p 5 –phred64’. Segregating polymorphic SNPs were called using the Samtools (v0.1.19) (Li *et al*., 2009a), and then filtered using VCFTools (0.1.19) (Danecek *et al*., 2011) with parameters of ‘‐d 0.8 –cvNg’, resulting in a total of 8525 SNP markers. These markers were adjusted to codes fit the software Joinmap^®^ 4.1 (Van Ooijen, 2006), filtered for low segregation distortion_._ Using the independence LOD method, 3664 markers were clustered into eight groups, with LOD scores ranging from 8 to 10, which equal the number of red bayberry chromosomes. The loci were ordered by the Regression Mapping Module, curated for spurious calls with graphical genotyping (Di Pierro *et al*., 2016) and recombination frequency threshold of 0.4 and an LOD threshold of 1.0. Map distances were calculated using Kosambi's mapping method, and 3075 SNPs were mapped on the eight linkage groups. Next, segregation information from partially informative single SNP markers from the same genetic bin was aggregated into highly informative HaploBlock markers (Di Pierro *et al*., 2016) with Haploblock Aggregator (https://www.wur.nl/en/product/HAPLOBLOCK-AGGREGATOR.htm). Genome‐wide recombination frequencies for all possible haploblock marker pairs were calculated in JoinMap^®^ and visualized by a heatmap made in R (Team, 2014).

The physical and genetic positions of the mapped markers were used to place and orient the female and male scaffolds and contigs relative to each other. Before pseudomolecule construction, a total of 3433 female contigs were compared against the male contigs using BLASTN with an *e*‐value cutoff of 1e^−25^. The integrated linkage map was used to construct female pseudomolecules (chromosomes). The map position of SNP markers aligned to anchor to the female scaffold. The orientation of the scaffolds with more than two SNPs was determined by the most common orientation indicated by all possible pairs of mapped markers when considering their order on the integrated genetic map. Based on the alignment between female and male genome, short and adjacent female scaffolds could be further ordered and extended based on the homology region. Adjacent scaffolds in each chromosome were separated by 1000 ‘N's. All unanchored contigs were connected randomly to chromosome 0.

### Gene prediction

The gene prediction pipeline of the female red bayberry genome combined *ab initio* gene prediction, homologous sequence searching and transcriptome sequence mapping. Glimmer HMM (v1.1.0) (Majoros *et al*., 2004), Fgenesh (v2.1) (Solovyev *et al*., 2006) and Augustus (v3.0.2) (Stanke *et al*., 2006) were used for *de novo* prediction. For homology‐based gene prediction, *A. thaliana*,* Glycine max*,* Oryza sativa*,* Prunus persica* and *Solanum lycopersicum* protein sequences were aligned to the *M. rubra* female genome using TBLASTN (Altschul *et al*., 1997), with an e‐value cutoff of 1e^−5^, followed by further alignments using GeneWise (v2.4.1) (Birney *et al*., 2004) for accurate exon‐intron information. GLEAN (v1.1) (Elsik *et al*., 2007) was used to merge *de novo* and homology‐based gene sets into the consensus gene set. Additionally, to identify the accurate splice junctions between each exon, the RNA‐seq data derived from eight of the ‘Y2012‐145’ libraries (Table S8) were first aligned to the female assembly using TopHat (Trapnell *et al*., 2010) (http://tophat.cbcb.umd.edu). On the basis of the alignments results, we employed Cufflinks (Trapnell *et al*., 2010) (v2.2.1) (http://cufflinks.cbcb.umd.edu) to obtain the open reading frames (ORFs) from the mapping result. Finally, we integrated GLEAN set with Cufflinks by in‐house pipeline (Kocher *et al*., 2013) to generate a final female gene set. For the male gene sets prediction, RNA‐seq data from three libraries of H2011‐12 (Table S8) were firstly aligned to the male assembly using TopHat (http://tophat.cbcb.umd.edu) and then the Cufflinks were used to get a set of assembled transcripts. The *de novo* annotated genes were named following the convention in apple (Daccord *et al*., 2017): *MR* (for *Morella rubra*) followed by the chromosome number (unmapped gene shown as 0) and gene number, for example *MR8G025877.1*.

### Phylogenetic tree and determination of speciation time

Red bayberry belongs to the Fagales of Eurosids I clade, a member of the Rosids clade. We select two genomes belong to Asterids including *Actinidia chinensis* (Kiwifruit) and *Solanum lycopersicum* (tomoto), and 10 belongs to Rosids including *Arabidopsis thaliana*,* Betula pendula*,* Carica papaya*,* Citrus sinensis*,* Figaria vesca*,* Juglans regia*,* Medicago truncatula*,* Morus notabilis*,* Populus trichocarpa*,* Prunus persica*,* Theobroma cacao*,* Vitis vinifera*. Comparative analysis was performed to establish the phylogenetic relationships of red bayberry at the genome‐wide level. Proteins from fifteen selected species were classified using all‐by‐all BLASTp, and OrhtoMCL (v2.0.2) (Li *et al*., 2003) was used to cluster the similar gene pairs into groups. Single‐copy orthologous gene clusters were extracted and used to construct the phylogenetic tree based on the maximum likelihood method by PhyML (Guindon and Gascuel, 2003) (v3.0). The MCMCTREE (v4.4) was used to estimate the divergence time of species using the Bayesian Relaxed Molecular Clock (BRMC) method, which is a part of the PAML package (Yang, 2007). The ‘Correlated molecular clock’ and ‘JC69’ model were selected. The MCMCTREE program was performed 100 000 times during speciation with a sample frequency of 2 and 10 000 iterations. The divergence time between *C. papaya* and *A. thaliana* of 54 to 90 Mya (Li *et al*., 2014) was used to calibrate the divergence time.

### Synteny analysis, whole‐genome duplication and evolution

The program MUMmer3.23 (http://mummer.sourceforge.net/) was used for synteny analysis of the female and male red bayberry genome, with parameters of ‘‐maxmatch chr1.fa lg1.fa’. The mapping results were filtered with the identity more than 85%. Graphics are shown using mummer plot.

We performed synteny searches to compare the red bayberry genome structure with peach and birch. To call syntenic blocks, we performed all‐by‐all BLASP with identity ≥40% and *e*‐value ≤1e‐10. Syntenic region within species were identified using MCScanx (Tang *et al*., 2008b) with parameters of ‘‐a ‐e 1e‐5 ‐u 10000 ‐s 5’. Based on the syntenic blocks, we performed synteny analysis of the relationship between *M. rubra*,* P. persica* and *Betula pendula*. To reconstruct the paleopolyploid history of red bayberry, we analysed the synteny among *M. rubra*,* M. truncatula*,* M. notabilis*,* J. regia*,* P. persica* and *B. pendula*. The 4‐fold degenerate third‐codon position (4DTv) of synteny blocks were calculated and revised by HKY (Hasegawa *et al*., 1985) model.

### Resequencing and sex‐specific region analysis

Bulked DNA libraries with 100 females and 100 males of *M. rubra* were sequenced giving 106‐fold and 127‐fold coverage respectively. Three main productive cultivars (Biqi, Dongkui and Xiazhihong) and three male plants (Y2015‐20, C2013‐14 and Y2010‐7) were resequenced to 36–58 fold coverage. To analysis the sex determination in red bayberry, we firstly mapped the two bulk reads (BSA‐F and BSA‐M) to the female reference assembly using SOAP with the parameter of ‘‐m 0 ‐x 1000 ‐s 40 ‐l 35 ‐v 7 ‐r 2’. SOAPsnp (Li *et al*., 2009b) was employed to identify the SNP of two bulks reads with the parameter of ‘‐u ‐t ‐L 150 ‐Q j’ and the depths of single base (SNPs) along the female reference genome were calculated for BSA‐F and BSA‐M by soap.coverage. To identify the female‐specific genome regions, the region with more than 100 consecutive base pair (>10 depth) BSA‐F reads were mapped and absence of the BSA‐M (0 depth) reads were obtained, a more than 50% coverage was considered as a candidate region. The candidate SNPs and insertions were checked in three individuals of each sex. The alignments of the candidate region with the female and male reference genomes were further processed. The sex‐linked markers developed, based on the female‐specific genes and their homologs, were tested in 133 female and 128 male individuals of *M. rubra* (Table S15). With the male (H2011‐12) genome reference, we also performed similar SNPs and Ins/Del linked to sex using two sex BSA reads and individuals.

## Data availability

The genome assemblies of female *M. rubra* Y2012‐145 and male *M. rubra* H2011‐12 have been deposited at GenBank: SAMN07510764 and SAMN07680263 respectively (Bio Project PRJNA398601).

## Authors’ contributions

Z.S.G., H.J.J. and Q.L.C. conceived the study and led the research together with J.Y.W. and G.Y.W. H.M.J. coordinated the sampling, bioinformatics and experimental work. Q.L.C. and J.L.G. performed the library construction and sequencing. D.L.Z., Q.L.C., J.Y.W. and J.L.G. assembled the genome, W.F.Y. and H.M.J. annotated the genome and analysed the sex‐specific region. H.M.J., Y.W., Y.T.S., H.B.Z., L.Z., H.B.X. and L.C. performed the DNA and RNA extraction. E.V.W and H.M.J. led the marker linkage mapping, assisted by W.Y., W.F.Y., X.W.L., H.J.J., Q.F.N. and H.B.Z. performed the RNA‐seq and qPCR. G.Y.W., C.Y.C., H.J.J., C.C.Z., Y.J. and T.T. were involved in establishing the mapping population and varieties maintenance and collection of the germplasm materials. W.F.Y., H.M.J. and J.Q. analysed the evolution and constructed the phylogenetic tree. H.B.Z., Y.W., L.Z. and X.W.L. tested the molecular markers in the germplasm. H.M.J., Z.S.G and H.J.J. drafted the paper with input from Q.L.C., E.V.W., W.Y., L.J.F., J.Y.W. and Y.H.L. All authors approved the final manuscript.

## Conflict of interest

Z.S.G., Y.W., H.M.J., H.B.Z., Y.J., C.Y.C. and G.Y.W. have two pending patents for the sex‐linked marker technique in China (201710221539.3 and 201710221205.6). The other authors declare they have no competing interests.

## Supporting information


**Figure S1** The female and male tree of red bayberry used for sequencing and assembly.
**Figure S2** The distribution of 17‐mer depth of the female and male Illumina PE reads.
**Figure S3** The red bayberry genetic haploblock (HB) map constructed using RAD tag sequencing technology
**Figure S4** Genome wide Heatmap of the recombination frequencies for 406 haploblocks from eight linkage groups of red bayberry.
**Figure S5** Integrated genetic and physical map of female red bayberry.
**Figure S6** Alignment of the female (a) and male (b) assembled scaffolds with the SNPs marker linkage genetic map.
**Figure S7** An example of assembly contigs with paired‐end relationship.
**Figure S8** Synteny between the female and male genome on eight pseudomolecules (chromosome) at 85% sequence identity.
**Figure S9** Area charts show quantification of retrotransposons (RT), DNA transposons (DNA‐TEs) and genes (both exons and introns) in eight chromosomes of female red bayberry.
**Figure S10** Divergence distribution of classified transposable element (TE) families in the M. rubra female genome.
**Figure S11** Tissue‐specific genes of the red bayberry.
**Figure S12** Distribution of basic genomic elements of red bayberry.
**Figure S13** Duplication events in the red bayberry genome.
**Figure S14** Venn diagram of orthologous gene families in five species.
**Figure S15** Location of the female‐specific region (FSR) by linkage mapping.
**Figure S16** Genome wide analysis of female‐specific insertions along the eight chromosomes with female reference.
**Figure S17** Genome wide analysis of male‐specific insertions along the eight chromosomes with male reference.
**Figure S18** Alignment of sequences of female‐specific gene *MrFT2* and its paralogous gene *MrFT1*.
**Figure S19** Amplification of sex‐linked marker derived from partial *McFT2* genes in American wax bayberry (*Morella cerifera)*.
**Figure S20** Overview of the processing pipeline used for the assembly of the red bayberry genome.
**Table S1** Summary of input sequence data for the assembly of female and male red bayberry genome.
**Table S2** Statistics of female and male *M. rubra* genome primary assembly.
**Table S4** Summary of genetic map of *M. rubra* from RAD‐sequencing of F_1_ population.
**Table S5** Summary of statistics of the transcriptome mapping to the red bayberry genome assembly.
**Table S6** The statement of the categories of BUSCO groups searched in red bayberry genome.
**Table S8** Tissue source for RNA‐seq and total amount of available sequence data.
**Table S9** General statistics for predicted protein‐coding genes for female red bayberry.
**Table S10** Functional annotation of predicted genes for female red bayberry.
**Table S12** Identification of non‐coding RNA genes in the red bayberry genome.
**Table S13** The statistics of gene families among different species.
**Table S16** Plant materials used for resequencing and BSA

**Table S3** The alignment results of two parents and their 95 progenies.
**Table S7** Classification of red bayberry repeat sequences.
**Table S11** Comparison of red bayberry transcription factors with other species in number observed per transcription factor class.
**Table S14** GO cluster/analysis for genes in *M. rubra* unique families.
**Table S15** Plant materials used for BSA sequencing, resequencing and sex‐specific primer PCR amplification.
**Table S17** The filtered SNP information in BSA and resequenced individuals.
**Table S18** Female‐specific genes and their paralogous on the same chromosome.
**Table S19** Primer sequences information.
