# Supplementary material for: The red bayberry genome and genetic basis of sex determination
Source: Plant Biotechnol J. 2018 Aug 10;17(2):397–409. doi: 10.1111/pbi.12985 (PMC6335074; doi:10.1111/pbi.12985)
Supplement: Supplementary file 1 — Figure S1 The female and male tree of red bayberry used for sequencing and assembly. Figure S2 The distribution of 17‐mer depth of the female and male Illumina PE reads. Figure S3 The red bayberry genetic haploblock (HB) map constructed using RAD tag sequencing technology Figure S4 Genome wide Heatmap of the recombination frequencies for 406 haploblocks from eight linkage groups of red bayberry. Figure S5 Integrated genetic and physical map of female red bayberry. Figure S6 Alignment of the female (a) and male (b) assembled scaffolds with the SNPs marker linkage genetic map. Figure S7 An example of assembly contigs with paired‐end relationship. Figure S8 Synteny between the female and male genome on eight pseudomolecules (chromosome) at 85% sequence identity. Figure S9 Area charts show quantification of retrotransposons (RT), DNA transposons (DNA‐TEs) and genes (both exons and introns) in eight chromosomes of female red bayberry. Figure S10 Divergence distribution of classified transposable element (TE) families in the M. rubra female genome. Figure S11 Tissue‐specific genes of the red bayberry. Figure S12 Distribution of basic genomic elements of red bayberry. Figure S13 Duplication events in the red bayberry genome. Figure S14 Venn diagram of orthologous gene families in five species. Figure S15 Location of the female‐specific region (FSR) by linkage mapping. Figure S16 Genome wide analysis of female‐specific insertions along the eight chromosomes with female reference. Figure S17 Genome wide analysis of male‐specific insertions along the eight chromosomes with male reference. Figure S18 Alignment of sequences of female‐specific gene MrFT2 and its paralogous gene MrFT1. Figure S19 Amplification of sex‐linked marker derived from partial McFT2 genes in American wax bayberry (Morella cerifera). Figure S20 Overview of the processing pipeline used for the assembly of the red bayberry genome. Table S1 Summary of input sequence data for the assembly of female and male re [file PBI-17-397-s002.docx]

**Supporting Information Appendix**

**The red bayberry genome and genetic basis of sex determination**

Hui-Min Jia^1, †^, Hui-Juan Jia^1, †^, Qing-Le Cai^2, †^, Yan Wang^1^, Hai-Bo Zhao^1^, Wei-Fei Yang^2^, Guo-Yun Wang^3^, Ying-Hui Li^4^, Dong-Liang Zhan^2^, Yu-Tong Shen^1^, Qing-Feng Niu^1,5^, Le Chang^1^, Jie Qiu^6^, Lan Zhao^1^, Han-Bing Xie^1^, Wan-Yi Fu^1^, Jing Jin^1^, Xiong-Wei Li^7^, Yun Jiao^8^, Chao-Chao Zhou^3^, Ting Tu^9^, Chun-Yan Chai^10^, Jing-Long Gao^2^, Long-Jiang Fan^6^, Eric van de Weg^11^, Jun-Yi Wang^2*^, Zhong-Shan Gao^1*^

^1^Institute of Fruit Science, College of Agriculture and Biotechnology, Zhejiang University, Hangzhou, 310058, China

^2^Hangzhou 1 Gene Ltd, Hangzhou, 310051, China

^3^Forestry Technology Extension Center, Yuyao, Ningbo 315400, China.

^4^The National Key Facility for Crop Gene Resources and Genetic Improvement (NFCRI), Institute of Crop Science, Chinese Academy of Agricultural Sciences, Beijing 100081, China

^5^Shanghai Center for Plant Stress Biology, and National Key Laboratory of Plant Molecular Genetics, Center of Excellence in Molecular Plant Sciences, Chinese Academy of Sciences, Shanghai, 200032, China

^6^Institute of Crop Science & Institute of Bioinformatics, College of Agriculture and Biotechnology, Zhejiang University, Hangzhou, 310058, China

^7^Forest & Fruit Tree Institute, Shanghai Academy of Agricultural Sciences, Shanghai, 201403, China

^8^Institute of Forestry, Ningbo Academy of Agricultural Science, Ningbo, China

^9^Shunmei Breeding and Propagation Centre for Chinese Bayberry, Yuyao, China

^10^Forestry Technology Extension Center, Cixi, 315300, China.

^11^Plant Breeding-Wageningen University and Research, P.O. Box 16, 6700AA, Wageningen, The Netherlands.

^12^Annoroad Gene Tech. Co., Ltd, Beijing, 100176, China

^†^These authors contributed equally to this work.

^*^Corresponding author: (Tel：+86 0571-88982225; FAX: +86 0571 88982224)

Email [gaozhongshan@zju.edu.cn](mailto:gaozhongshan@zju.edu.cn) (ZSG) or [wangjunyi@1gene.com.cn](mailto:wangjunyi@1gene.com.cn) (JYW)

[1. Sequencing and assembly of the red bayberry genome 5](#_Toc518226653)

[1.1 plant materials 5](#_Toc518226654)

[1.2 Nuclear DNA preparation. 5](#_Toc518226655)

[1.3 Library constructions 5](#_Toc518226656)

[1.5 *De novo* assembly of the red bayberry genome 7](#_Toc518226657)

[2. Genome annotation 8](#_Toc518226658)

[1.4 Genome size estimation 8](#_Toc518226659)

[2.1 Repetitive sequences 8](#_Toc518226660)

[2.2 Gene prediction and annotation 9](#_Toc518226661)

[2.3 Identification of non-coding RNA genes 9](#_Toc518226662)

[3. Analysis of Sex determination in red bayberry. 9](#_Toc518226663)

[3.1 BSA (Bulked segregant analysis) approach 10](#_Toc518226664)

[3.2 Resequencing 10](#_Toc518226665)

[3.2 Cloning the gap between S_5204 and S_2925a 10](#_Toc518226666)

[4. Supplemental Reference 11](#_Toc518226667)

[5. Supplementary Figures and legends 14](#_Toc518226668)

[Figure S1. The female and male tree of red bayberry used for sequencing and assembly. 14](#_Toc518226669)

[Figure S2. The distribution of 17-mer depth of the female and male Illumina PE reads. 15](#_Toc518226670)

[Figure S3. The red bayberry genetic haploblock (HB) map constructed using RAD tag sequencing technology. 16](#_Toc518226671)

[Figure S4. Genome wide Heatmap of the recombination frequencies for 406 haploblocks from eight linkage groups of red bayberry. 17](#_Toc518226672)

[Figure S5. Integrated genetic and physical map of female red bayberry. 18](#_Toc518226673)

[Figure S6. Alignment of the female (a) and male (b) assembled scaffolds with the SNPs marker linkage genetic map. 19](#_Toc518226674)

[Figure S7. An example of assembly contigs with paired-end relationship. 20](#_Toc518226675)

[Figure S8. Synteny between the female and male genome on eight pseudomolecules (chromosome) at 85% sequence identity. 21](#_Toc518226676)

[Figure S9. Area charts show quantification of retrotransposons (RT), DNA transposons (DNA-TEs) and genes (both exons and introns) in eight chromosomes of female red bayberry. 22](#_Toc518226677)

[Figure S10. Divergence distribution of classified transposable element (TE) families in the M. rubra female genome. 23](#_Toc518226678)

[Figure S11. Tissue-specific genes of the red bayberry. 24](#_Toc518226679)

[Figure S12. Distribution of basic genomic elements of red bayberry. 25](#_Toc518226680)

[Figure S13. Duplication events in the red bayberry genome. 26](#_Toc518226681)

[Figure S14. Venn diagram of orthologous gene families in five species. 27](#_Toc518226682)

[Figure S15. Location of the female specific region (FSR) by linkage mapping. 28](#_Toc518226683)

[Figure S16. Genome wide analysis of female-specific insertions along the eight chromosomes with female reference. 29](#_Toc518226684)

[Figure S17. Genome wide analysis of male-specific insertions along the eight chromosomes with male reference. 30](#_Toc518226685)

[Figure S18. Alignment of sequences of female-specific gene *MrFT2* and its paralogous gene *MrFT1*. 31](#_Toc518226686)

[Figure S19. Amplification of sex-linked marker derived from partial *McFT2* genes in American wax bayberry (*Morella cerifera)*. 32](#_Toc518226687)

[Figure S20. Overview of the processing pipeline used for the assembly of the red bayberry genome. 33](#_Toc518226688)

[6. Supplementary Table and legends 34](#_Toc518226689)

[Table S1. Summary of input sequence data for the assembly of female and male red bayberry genome 34](#_Toc518226690)

[Table S2. Statistics of female and male *M. rubra* genome primary assembly. 35](#_Toc518226691)

[Table S3. The alignment results of two parents and their 95 progenies 36](#_Toc518226692)

[Table S4. Summary of genetic map of *M. rubra* from RAD-sequencing of F_1_ population. 36](#_Toc518226693)

[Table S5. Summary of statistics of the transcriptome mapping to the red bayberry genome assembly. 37](#_Toc518226694)

[Table S6. The statement of the categories of BUSCO groups searched in red bayberry genome 38](#_Toc518226695)

[Table S7. Classification of red bayberry repeat sequences 38](#_Toc518226696)

[Table S8. Tissue source for RNA-seq and total amount of available sequence data 39](#_Toc518226697)

[Table S9. General statistics for predicted protein-coding genes for female red bayberry. 40](#_Toc518226698)

[Table S10. Functional annotation of predicted genes for female red bayberry. 41](#_Toc518226699)

[Table S11. Comparison of red bayberry transcription factors with other species in number observed per transcription factor class 41](#_Toc518226700)

[Table S12. Identification of non-coding RNA genes in the red bayberry genome. 42](#_Toc518226701)

[Table S13. The statistics of gene families among different species. 43](#_Toc518226702)

[Table S14. GO cluster/analysis for genes in *M. rubra* unique families 44](#_Toc518226703)

[Table S15. Plant materials used for BSA sequencing, resequencing and sex specific primer PCR amplification 44](#_Toc518226704)

[Table S16. Plant materials used for resequencing and BSA. 44](#_Toc518226705)

[Table S17. The filtered SNP information in BSA and re-sequenced individuals 44](#_Toc518226706)

[Table S18. Female specific-genes and their paralogous on the same chromosome 8 44](#_Toc518226707)

[Table S19. Primer sequences information 44](#_Toc518226708)

# 1. Sequencing and assembly of the red bayberry genome

## 1.1 plant materials

*Morella rubra* is a subtropical dioecious tree (usually known as red bayberry), female plants bear fruits, and male plants used to pollinate (Figure S1). The female red bayberry individual ‘Y2012-145’ was used for reference female genome sequencing because of its lower heterozygosity ([Jia et al., 2014](#_ENREF_11)). It is derived from a seedling line of ‘Shuijing’ with light yellow fruit color, about 60-year old, growing in the farm of Yuyao, Ningbo city, Zhejiang Province, China. The male individual ‘H2011-12’, used for male reference genome sequencing grows in Hangzhou, Zhejiang Province.

## 1.2 Nuclear DNA preparation.

Fresh leaves from red bayberry were collected, and nuclear DNA was isolated following the procedure described previously ([Jia et al., 2015](#_ENREF_10)). DNA purity and quantity was determined by NanoDrop^TM^ Spectrophotometers (Thermo Scientific)

## 1.3 Library constructions

**1.3.1 Genomic libraries**

**1.3.1.1 Illumina paired-end libraries and sequencing**

For the female (Y2012-145) genome, standard protocol of construction of Illumina paired-end genome library, seven paired-end Illumina WGS libraries were constructed with multiple insert sizes (200bp, 500bp, 800bp, 2 kb, 5 kb, 10 kb and 20 kb) according to the manufacturer’s instructions (Illumina), and then sequenced on Illumina HiSeq 4000 system . Finally，we collected a total clean data amount of 91.9 Gb. For the male (H2011-12) genome, a paired-end library with 450 bp insert size was constructed and sequenced with PE250, which generated a total of 15.7 Gb clean data (Table S1). The above two genomes sequencing were generated by 1Gene Ltd, Hangzhou, China.

**1.3.1.2 Pacbio libraries and sequencing**

Library construction for PacBio sequencing was carried out using the protocols recommended by the manufacturer. We constructed 20 kb single-molecule reads library for female individual ‘Y2012-145’ and male individual ‘H2011-12’ respectively, and then sequenced on a PacBio RSII Sequencer using P6/C4 chemistry system, and generated total 5.1 Gb and 19.7 Gb clean data for female and male genome respectively, all sequencing were performed at the 1Gene Ltd, Hangzhou, China (Table S1).

**1.3.2 RNA libraries**

**1.3.2.1 Plant material**

Red bayberry RNA library tissues (Table S8) were obtained from ‘Y2012-145’ and ‘H2011-12’. Root, stem, young leaves, buds, female flowers and very young fruits 20 days after pollination (DAP) were collected from the female line ‘Y2012-145’ (Figure S1). The Young fruit (43 DAP), break fruit (54 DAP) and ripe fruit (66 DAP) of the female line ‘Y2012-145’ (slightly pink fruit varieties) were sampled on June 1st, 11 th, and 19 th, 2015, respectively, with three biological replicates. Male buds and flowers at different development stage were collected from the male individual ‘H2011-12’ (Table S8). After sample collection, tissues were frozen immediately in liquid nitrogen and stored in -80 ˚С until RNA extraction.

**1.3.2.2 Illumina RNA sequencing**

Total RNA was isolated from different Chinese bayberry tissues using the CTAB method and purified by phenol/chloroform extraction then checked using an Agilent 2100 Bioanalyzer. Total RNA was processed in preparation for Illumina sequencing, according to the previous report. The constructed libraries were sequenced on Illumina Hiseq 4000 sequencer (150 bp paired-end reads). The cleaned RNA-seq reads were firstly aligned to the Chinese bayberry genome assembly using TopHat (v2.0.11), then the reads counts mapped to the genome of each bayberry gene model and normalized to fragments per kilobase of exon model per million mapped reads (RPKM).

For gene expression analysis, first-strand cDNA was synthesized from 1μg total RNA using the PrimeScript™ RT reagent Kit with gDNA Eraser (Takara, Kyoto, Japan) and qPCR was performed with SYBR Premix Ex Taq (Takara) according to the manufacturer’s instructions. The measurements of the gene expression were obtained using the relative quantification method and the relative expression level was normalized to that of the actin gene. The data was analyzed by 2-ΔΔCt method. The primers for qPCR are listed in Table S19.

## 1.5 *De novo* assembly of the red bayberry genome

The red bayberry Genome assembly pinepline is shown in Figure S20. In summary, two intermediate assembly versions for the genome were generated using Illumina reads (v0.1) by SOAPdenovo2 ([Luo et al., 2012](#_ENREF_16)) with default parameters and PacBio reads (v0.2) by Falcon ([Chin et al., 2016](#_ENREF_5)), separately. Then they were merged together using the HABOT ([Zou et al., 2017](#_ENREF_27)) software and then the gap filling was performed using Illumina reads to obtain the final assembly v1.0. To combine contigs from different versions of assembly and construct a new contig sets, HABOT with the following four modules were used: firstly, Graph module. This module counts k-mer frequencies and extracts the unique k-mers from Illumina reads. A unique k-mer is theoretically defined as k-bp sequences that occur just once in a haploid genome and is calculated following a Poisson model as previously described ([You et al., 2013](#_ENREF_24)). Using unique k-mers, instead of all the k-mers, for graph construction minimizes the effects of error-prone repeats and increases computation speed. Secondly, align module. This module is use for an all-to-all alignment between PacBio contigs and Illumina contigs. By using unique k-mers it performs the alignment much faster than BLASR and is of high accuracy. Thirdly, Duplication remove module. When two sequences have common uinque k-mers that exceed a cutoff (default is 0.5), the shorter sequence is removed. The last step is *de novo* module. This module calls the above 3 modules and performs hybrid assembly.

For the assembly, we extracted unique 17-mers from the two PCR-free Illumina reads. Overlaps among contigs from different intermediate assembly versions were identified the alignment module. Then the OLC graph was built by the overlap contigs. The connection is dropped in case of the following situation: (1) contig A’s best connection is contig B; (2) contig B’s best connection is contig C; (3) give up connection from A to B if A has no alignment with C. Afterwards duplicated regions in the contig set were removed. Finally scaffolding and gap closure were performed on the new contig set using Illumina mate pair reads with SSPACE ([Boetzer et al., 2011](#_ENREF_4)) v3.0 and GapCloser ([Luo et al., 2012](#_ENREF_16)) v1.12 (both with default parameters) to generate assembly v1.0. Finally we got the final assembly version for female genome with genome size of 312.7 Mb and 313.5 Mb for male genome.

We provide a simplified version of HABOT in self-build galaxy service (http://61.130.10.147:9010/). One should contact the author for a new account to use it.

# 2. Genome annotation

## 1.4 Genome size estimation

K-mer refer to a sequence with k nucleotides was often used to estimate the size of genome. The raw Illumina reads were filtered for PCR duplicates, low-quality reads, adaptor sequences before genome assemble. Total of 18.9 Gb clean data from 500 bp-insert size female library and 48.7 Gb clean data from 250 bp-insert size male library were used to perform the K-mer analysis by dividing short reads into 17 nt and calculating the frequency of each k-mer (Figure S2). The k-mer frequency follows a Poisson distribution beyond a certain quantity of data, allowing the use of this information to estimate the genome size, as well as to inspect the heterozygosity rate and repeat content. The followed formula were conducted to calculate the genome size:“genome size =k_num/Peak_depth,” where k_num is the total number of k-mers and Peak_depth is the expected value of k-mer depth. Generally, k value is 17, as is the case in the k-mer analysis of Y2012-145. The peak depth is 51 and k-mer number was 16,456,663,652. Thus, the Y2012-145 genome size was estimated to be 322.68 Mb which is consistent with earlier red bayberry male genome size 323 Mb obtained by using whole genome shotgun sequencing ([Jiao et al., 2012](#_ENREF_12)). While the H2011-12 male genome size was estimated to be 319.2 Mb. Analysis of the 17-mer frequency distribution based on short insert size clean data revealed the heterozygosity of female was 0.56% and male 0.70% (Figure S2), shows a low and medium heterozygosity among the sequenced diploid perennial wood plants such as kiwifruit (0.54%) ([Huang et al., 2013](#_ENREF_9)), *E. ulmoides* (0.8%) ([Wuyun et al., 2018](#_ENREF_21)), pear (1.0%) ([Wu et al., 2013](#_ENREF_20)) and jujube (1.9%) ([Liu et al., 2014](#_ENREF_14)).

## 2.1 Repetitive sequences

Repeat sequences of female bayberry genome were identified with a combination of *de novo* and homolog strategies. RepeatMasker (v4.0.3) and RepeatProteinMask (3.3.0) (<http://www.repeatmasker.org>) software were employed to find the transposons components based on the RepBase library (<http://www.girinst.org/repbase>) with an e-value cutoff 1*e*^-5^. TRF ([Benson, 1999](#_ENREF_3)) (4.07b) software was used to identify tandem repeats. Piler ([Edgar and Myers, 2005](#_ENREF_7)) (v1.0) and LTR-FINDER ([Xu and Wang, 2007](#_ENREF_23)) (v1.05) were used to build *de novo* repeat sequences in the assemble genome and RepeatMasker was used to identify positions of repeats. Finally, the identified repeat sequences were used to construct a non-redundant repeat sequence library, and repeat sequences with an identity more than 50% were grouped into the same classes. The female and male genome had 114 Mb and 154.6 Mb repetitive sequences, accounting for 36.4% and 49.3% of the assembly. The proportion of repetitive sequences in the bayberry genome is higher than peach ([Verde et al., 2013](#_ENREF_19)) and poplar ([Tuskan et al., 2006](#_ENREF_18)), comparable to that in mulberry ([He et al., 2013](#_ENREF_8)) (47%) silver birch ([Salojarvi et al., 2017](#_ENREF_17)) (49.2%) and pomegranate ([Yuan et al., 2018](#_ENREF_25)) (51.2%), and lower than that in pear (53.1%) ([Wu et al., 2013](#_ENREF_20)), apple (57.3%) ([Daccord et al., 2017](#_ENREF_6)) and tea tree (80.9%) ([Xia et al., 2017](#_ENREF_22)).

## 2.2 Gene prediction and annotation

Annotation of the predicted genes was performed by blasting their sequences against InterPro ([Zdobnov and Apweiler, 2001](#_ENREF_26)), Gene Ontology ([Ashburner et al., 2000](#_ENREF_1)), KEGG ([Kanehisa and Goto, 2000](#_ENREF_13)), Swissprot and TREMBL ([Bairoch and Apweiler, 2000](#_ENREF_2)) database with an *e*-value cutoff of 1e-5. Nearly 90% of genes can be annotated in known database (Table S10). The gene density, repeat sequences, SNPs, SSRs and GC content distributions along the female pseudo-chromosomes are illustrated (Figure S12). A total of 724,977 SNPs were identified with a density of 2.2 SNPs per Kb, unevenly distributed across the female chromosomes, with more in chromosomes 1, 2 and 8. The 41,791 SSR loci (0.13 per Kb) were evenly distributed (Figure S12).

## 2.3 Identification of non-coding RNA genes

The non-coding RNA were identified by searching the genome assembly against the Rfam11.0 database using INFERNAL (v1.1) with default parameters (<http://infernal.janelia.org>/). tRNA were identified by using tRNAscan-SE ([Lowe and Eddy, 1997](#_ENREF_15)), we also identified rRNA, tRNA, miRNA and snRNA. Finally, we identified a total of 626 tRNA, 128 miRNAs and 489 rRNA (Table S12).

Transcription factors (TFs) were identified and classified into different families using the PlnTFDB (<http://plntfdb.bio.uni-potsdam.de/v3.0/>) to query red bayberry proteome. A total of 1387 putative red bayberry TFs distributed in 58 families, have been identified (Table S11).

# 3. Analysis of Sex determination in red bayberry.

## 3.1 BSA (Bulked segregant analysis) approach

High-quality genomic DNA was extracted from the 100 female plants and 100 male plants of *M. rubra* and pooled as female bulked DNA (BSA-F) and male bulked DNA (BSA-M), in an equal ration (Table S15). Libraries of insert size of 300 bp were generated for each bulk and sequenced on the Illumina HiSeq 2000 sequencer (150 bp paired-end reads). A total of 34.4 Gb and 41 Gb clean read were generated with 106x and 127x coverage for BSA-F and BSA-M, respectively (Table S16).

## 3.2 Resequencing

Three main productive cultivars (Biqi, Dongkui and Xiazhihong) and three male individuals including Y2015-20, C2013-14 and Y2010-7 were sampled for resequencing to analysis the sex determination mechanism as well as genetic diversity of main cultivars. The Illumina paired-end library were constructed for three female individual with 500bp insert size, and sequenced on HiSeq 4000 platform and yielding 18 Gb, 12 Gb and 11 Gb clean data as paired 150 bp reads respectively. Four male Illumina paired-end libraries with target insert 450bp were constructed and sequenced on HiSeq 2500 platform with PE250 (Table S16).

## 3.2 Cloning the gap between S_5204 and S_2925a

The nested PCR was used to identify the gap between S_5204 and S_2925a, the primer was designed based on the alignment between female S_906a and S_5204, female S_906a and S_2925a. The primers are listed in Table S19. PCR products were analyzed on 1% agarose gels. For each reaction product, a single fragment was recovered from the gels and purified using a DNA purification kit (Takara). The fragment was then ligated into the pEASY-Blunt Simple vector, transformed into E. coli DH5α competent cells (TRANSGENE BIOTECH), and then sequenced (Sangong, Shanghai, China).

# 4. Supplemental Reference

Ashburner, M., Ball, C.A., Blake, J.A., Botstein, D., Butler, H., Cherry, J.M., Davis, A.P., Dolinski, K., Dwight, S.S., Eppig, J.T., et al. (2000). Gene Ontology: tool for the unification of biology. Nature Genetics 25:25-29.

Bairoch, A., and Apweiler, R. (2000). The SWISS-PROT protein sequence database and its supplement TrEMBL in 2000. Nucleic Acids Research 28:45-48.

Benson, G. (1999). Tandem repeats finder: a program to analyze DNA sequences. Nucleic Acids Research 27:573-580.

Boetzer, M., Henkel, C.V., Jansen, H.J., Butler, D., and Pirovano, W. (2011). Scaffolding pre-assembled contigs using SSPACE. Bioinformatics 27:578-579.

Chin, C.S., Peluso, P., Sedlazeck, F.J., Nattestad, M., Concepcion, G.T., Clum, A., Dunn, C., O'Malley, R., Figueroa-Balderas, R., Morales-Cruz, A., et al. (2016). Phased diploid genome assembly with single-molecule real-time sequencing. Nature Methods 13:1050.

Daccord, N., Celton, J.-M., Linsmith, G., Becker, C., Choisne, N., Schijlen, E., van de Geest, H., Bianco, L., Micheletti, D., Velasco, R., et al. (2017). High-quality *de novo* assembly of the apple genome and methylome dynamics of early fruit development. Nature Genetics 49:1099-1116.

Edgar, R.C., and Myers, E.W. (2005). PILER: identification and classification of genomic repeats. Bioinformatics 21:i152-i158.

He, N., Zhang, C., Qi, X., Zhao, S., Tao, Y., Yang, G., Lee, T.-H., Wang, X., Cai, Q., and Li, D. (2013). Draft genome sequence of the mulberry tree *Morus notabilis*. Nature Communications 4:2445.

Huang, S., Ding, J., Deng, D., Tang, W., Sun, H., Liu, D., Zhang, L., Niu, X., Zhang, X., and Meng, M. (2013). Draft genome of the kiwifruit *Actinidia chinensis*. Nature Communications 4:2640.

Jia, H.M., Jiao, Y., Wang, G.Y., Li, Y.H., Jia, H.J., Wu, H.X., Chai, C.Y., Dong, X., Guo, Y.P., Zhang, L.P., et al. (2015). Genetic diversity of male and female Chinese bayberry (*Myrica rubra*) populations and identification of sex-associated markers. BMC Genomics 16:394.

Jia, H.M., Shen, Y.T., Jiao, Y., Wang, G.Y., Dong, X., Jia, H.J., Du, F., Liang, S.M., Zhou, C.C., and Mao, W.H. (2014). Development of 107 SSR markers from whole genome shotgun sequences of Chinese bayberry (*Myrica rubra*) and their application in seedling identification. Journal of Zhejiang University Science B 15:997-1005.

Jiao, Y., Jia, H.M., Li, X.W., Chai, M.L., Jia, H.J., Chen, Z., Wang, G.Y., Chai, C.Y., Van de Weg, E., and Gao, Z.S. (2012). Development of simple sequence repeat (SSR) markers from a genome survey of Chinese bayberry (*Myrica rubra*). BMC Genomics 13:201.

Kanehisa, M., and Goto, S. (2000). KEGG: Kyoto Encyclopedia of Genes and Genomes. Nucleic Acids Research 28:27-30.

Liu, M.J., Zhao, J., Cai, Q.-L., Liu, G.-C., Wang, J.-R., Zhao, Z.-H., Liu, P., Dai, L., Yan, G., and Wang, W.-J. (2014). The complex jujube genome provides insights into fruit tree biology. Nature communications 5:5315.

Lowe, T.M., and Eddy, S.R. (1997). tRNAscan-SE: a program for improved detection of transfer RNA genes in genomic sequence. Nucleic Acids Research 25:955-964.

Luo, R., Liu, B., Xie, Y., Li, Z., Huang, W., Yuan, J., He, G., Chen, Y., Pan, Q., and Liu, Y. (2012). SOAPdenovo2: an empirically improved memory-efficient short-read de novo assembler. Gigascience 1:18.

Salojarvi, J., Smolander, O.P., Nieminen, K., Rajaraman, S., Safronov, O., Safdari, P., Lamminmaki, A., Immanen, J., Lan, T.Y., Tanskanen, J., et al. (2017). Genome sequencing and population genomic analyses provide insights into the adaptive landscape of silver birch. Nature Genetics 49:904-912.

Tuskan, G.A., Difazio, S., Jansson, S., Bohlmann, J., Grigoriev, I., Hellsten, U., Putnam, N., Ralph, S., Rombauts, S., and Salamov, A. (2006). The genome of black cottonwood, *Populus trichocarpa* (Torr. & Gray). Science 313:1596-1604.

Verde, I., Abbott, A.G., Scalabrin, S., Jung, S., Shu, S., Marroni, F., Zhebentyayeva, T., Dettori, M.T., Grimwood, J., and Cattonaro, F. et al. (2013). The high-quality draft genome of peach (*Prunus persica*) identifies unique patterns of genetic diversity, domestication and genome evolution. Nature Genetics 45:487-494.

Wu, J., Wang, Z., Shi, Z., Zhang, S., Ming, R., Zhu, S., Khan, M.A., Tao, S., Korban, S.S., Wang, H., et al. (2013). The genome of the pear (*Pyrus bretschneideri* Rehd.). Genome Research 23:396-408.

Wuyun, T.N., Wang, L., Liu, H., Wang, X., Zhang, L., Bennetzen, J.L., Li, T., Yang, L., Liu, P., Du, L., et al. (2018). The Hardy Rubber Tree Genome Provides Insights into the Evolution of Polyisoprene Biosynthesis. Molecular Plant 11:429-442.

Xia, E.-H., Zhang, H.-B., Sheng, J., Li, K., Zhang, Q.-J., Kim, C., Zhang, Y., Liu, Y., Zhu, T., Li, W., et al. (2017). The Tea Tree Genome Provides Insights into Tea Flavor and Independent Evolution of Caffeine Biosynthesis. Molecular Plant 10:866-877.

Xu, Z., and Wang, H. (2007). LTR_FINDER: an efficient tool for the prediction of full-length LTR retrotransposons. Nucleic Acids Research 35:W265-W268.

You, M., Yue, Z., He, W., Yang, X., Yang, G., Xie, M., Zhan, D., Baxter, S.W., Vasseur, L., and Gurr, G.M. (2013). A heterozygous moth genome provides insights into herbivory and detoxification. Nature Genetics 45:220-225.

Yuan, Z., Fang, Y., Zhang, T., Fei, Z., Han, F., Liu, C., Liu, M., Xiao, W., Zhang, W., Wu, S., et al. (2018). The pomegranate (*Punica granatum* L.) genome provides insights into fruit quality and ovule developmental biology. Plant Biotechnology Journal 16:1363-1374.

Zdobnov, E.M., and Apweiler, R. (2001). InterProScan–an integration platform for the signature-recognition methods in InterPro. Bioinformatics 17:847-848.

Zou, C.S., Chen, A.J., Xiao, L.H., Muller, H.M., Ache, P., Haberer, G., Zhang, M.L., Jia, W., Deng, P., Huang, R., et al. (2017). A high-quality genome assembly of quinoa provides insights into the molecular basis of salt bladder-based salinity tolerance and the exceptional nutritional value. Cell Research 27:1327-1340.

# 5. Supplementary Figures and legends


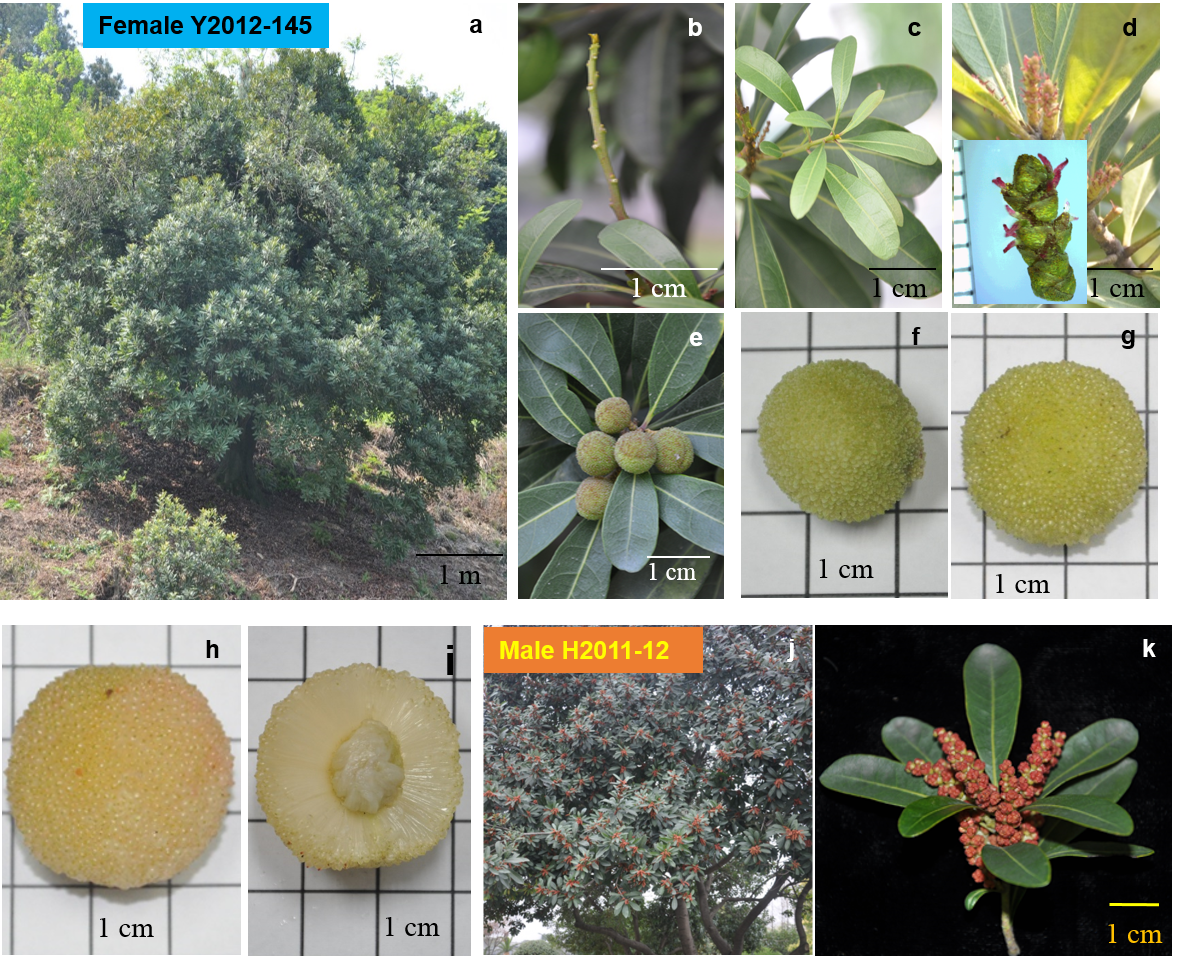


Figure S1. The female and male tree of red bayberry used for sequencing and assembly. a- Female red bayberry tree Y2012-145; b-stem; c-leaf; d-female flower; e- small fruit; f, g, h and i are three fruit developing and mature stages; j- male tree of H2011-12; k- male flower.

Figure S2. The distribution of 17-mer depth of the female and male Illumina PE reads. The analyzed reads from female and male libraries with insert sizes of 500 bp and 450bp after filtering raw reads and sequence error correction, respectively. According to *K-mer* frequency information, the peak depth is 51 and 45, and the genome size of female and male *M. rubra* were estimated as 323 Mb and 319 Mb, respectively

## Figure S3. The red bayberry genetic haploblock (HB) map constructed using RAD tag sequencing technology.

The HB markers lm x ll shown in red, nn x np in green, and ab x cd segregation type is in blue. The HBs in centromere are indicated in bold.

## Figure S4. Genome wide Heatmap of the recombination frequencies for 406 haploblocks from eight linkage groups of red bayberry.

## Figure S5. Integrated genetic and physical map of female red bayberry.

Physical distance along the indicated pseudomolecules is on the vertical axis (Mb), and genetic distance on the horizontal axis (centiMorgan, cM). The genetic map was constructed using a F_1_ population from the ‘Biqi’ and ‘Dongkui’ cross. The FSR (female specific region) was located on the distal end of pseudo-chromosome 8.

## Figure S6. Alignment of the female (a) and male (b) assembled scaffolds with the SNPs marker linkage genetic map.

Figure S7. An example of assembly contigs with paired-end relationship. Medium orchid, lime green, green, blue, red, and black lines represent paired-end reads with insert sizes of 200, 500, 2000, 5000, 10000, and 20000 bp, respectively.

Figure S8. Synteny between the female and male genome on eight pseudomolecules (chromosome) at 85% sequence identity.

## Figure S9. Area charts show quantification of retrotransposons (RT), DNA transposons (DNA-TEs) and genes (both exons and introns) in eight chromosomes of female red bayberry.

Figure S10. Divergence distribution of classified transposable element (TE) families in the M. rubra female genome. The classified TE families in the *M. rubra* genome were aligned to the consensus sequences in the Repbase library. The sequence divergence rates of TEs were counted.

## Figure S11. Tissue-specific genes of the red bayberry.

## Figure S12. Distribution of basic genomic elements of red bayberry.

a, synteny relationship of gene blocks between pseudo-chromosomes; b, ideograms of the eight pseudo-chromosomes; c, GC content density; d, SNP density; e, SSR density; f, repeat density and g, gene density.

## Figure S13. Duplication events in the red bayberry genome.

4DTv (fourfold synonymous third-codon transversion) value between syntenic gene pairs in red bayberry, mulberry (*Morus notabilis*), peach (*Prunus persica*) medicago (*Medicago truncatula*), silver birch (*Betula pendula*), walnut (*Juglans regia*), poplar (*Populus trichocarpa)*, papaya (*Carica papaya)* and *Arabidopsis thaliana*.

Figure S14. Venn diagram of orthologous gene families in five species. *Morella rubra*, *Populus trichocarpa*, *Morus notabilis*, *Carica papaya*, and *Arabidopsis thaliana* were used to generate the Venn diagram based on gene family cluster analysis (OrthoMCL E<1e-5).

## Figure S15. Location of the female specific region (FSR) by linkage mapping.

Quality control on the genetic position of two sex linked SCAR markers based on co-segregation patterns in the maternal (A) and paternal parent (B) in 95 individuals of the mapping population using Graphical Genotyping. Markers are in rows, individuals in columns. Recombination events are characterized by transitions in filled colour within a column (blue/yellow) or a blank/blue or blank/yellow patterns resulting from a combination of the two common pattern of that colour type across all individuals. Sites of recombination are marked by two flanking pink bordered genotype calls. Red bordered cells indicate spurious double recombination events that are mostly due to erroneous calling as they could not be solved by alternative marker ordering. Blue lines mark the transition between two flanking haploblocks. Grey filled cells mark missing genotype calls.

Figure S16. Genome wide analysis of female-specific insertions along the eight chromosomes with female reference. Red dots represent female reads with more than 100 consecutive base pairs mapped on the female chromosome. Absence of male reads shown as blue dots.

Figure S17. Genome wide analysis of male-specific insertions along the eight chromosomes with male reference. Blue dots represent male reads with more than 100 consecutive base pairs mapped on the male chromosome. Absence of female reads shown with red dots.

Figure S18. Alignment of sequences of female-specific gene *MrFT2* and its paralogous gene *MrFT1*. The sex-linked marker sequences are shaded in green and yellow.

## Figure S19. Amplification of sex-linked marker derived from partial *McFT2* genes in American wax bayberry (*Morella cerifera)*.

## Figure S20. Overview of the processing pipeline used for the assembly of the red bayberry genome.

# 6. Supplementary Table and legends

## Table S1. Summary of input sequence data for the assembly of female and male red bayberry genome

| **Accession** | **Library** | **Insert Size** | **Average Reads Length(bp)** | **Data after filtering (Gb)** | **Coverage (×)** |
| --- | --- | --- | --- | --- | --- |
|  |  |  |  |  |  |
| Y2012-145 (Female) | Illumina PE 1 | 200bp | 125 | 18.6 | 56 |
|  | Illumina PE 2 | 500bp | 125 | 18.87 | 57 |
|  | Illumina PE 3 | 800bp | 150 | 3.98 | 12 |
|  | Illumina PE 4 | 2kb | 150 | 16.72 | 51 |
|  | Illumina PE 5 | 5kb | 150 | 20.65 | 63 |
|  | Illumina PE 6 | 10kb | 150 | 3.34 | 10 |
|  | Illumina PE 7 | 20kb | 150 | 9.72 | 29 |
|  | PacBio reads | 20kb | 11,574 | 5.11 | 15 |
| Total |  | - |  | 96.99 | 293 |
| H2011-12 (Male) | Illumina PE 1 | 450bp | 250 | 15.70 | 48 |
|  | PacBio reads | 20k | 6,841 | 19.70 | 61 |
| Total |  | - |  | 35 | 109 |

## Table S2. Statistics of female and male *M. rubra* genome primary assembly.

| **Genome features** | **Female genome** | | | | **Male genome** | |
| --- | --- | --- | --- | --- | --- | --- |
|  | **Scaffold** | | **Contig** | | **scaffold** | |
|  | **Size (bp)** | **Number** | **Size (bp)** | **Number** | **Size (bp)** | **Number** |
| Longest | 6,316,743 | - | 1,664,236 | - | 5,559,420 | - |
| N10 | 2,632,994 | 9 | 620,512 | 35 | 3,238,249 | 8 |
| N20 | 1,660,650 | 25 | 398,298 | 98 | 2,208,872 | 20 |
| N30 | 1,206,295 | 47 | 297,152 | 189 | 1,823,546 | 35 |
| N40 | 837,221 | 78 | 234,298 | 306 | 1,427,556 | 55 |
| N50 | 635,176 | 121 | 192,565 | 450 | 1,135,429 | 80 |
| N60 | 482,718 | 176 | 153,603 | 628 | 854,703 | 111 |
| N70 | 368,374 | 250 | 118,132 | 854 | 612,878 | 155 |
| N80 | 281,418 | 346 | 87,542 | 1156 | 331,401 | 220 |
| N90 | 177,695 | 484 | 55,104 | 1594 | 38,316 | 509 |
| Total_length | 312,683,233 | - | 306,859,782 | - | 313507419 | - |
| number≥100bp | - | 1,114 | - | 3,433 |  | 8602 |
| number≥2000bp | - | 962 | - | 2,939 |  | 3750 |

Table S3. The alignment results of two parents and their 95 progenies (in Excel file).

## Table S4. Summary of genetic map of *M. rubra* from RAD-sequencing of F_1_ population.

| **Linkage group** | **Marker number** | **Total genetic disance(cM)** | **Average genetic distance (cM)** | **Haploblock Bin Number** | **Centromere region in linkage map** | **Centromere region length Mb** | **Total anchored length of Female (bp)** | **Total anchored length of Male (bp)** |
| --- | --- | --- | --- | --- | --- | --- | --- | --- |
| LG1 | 466 | 99.5 | 0.21 | 62 | 60-62 | 6.2 | 38,479,963 | 37,736,660 |
| LG2 | 425 | 63.6 | 0.15 | 49 | 42-44 | 8.6 | 39,591,731 | 36,603,842 |
| LG3 | 387 | 53.8 | 0.14 | 50 | 42-44 | 12.7 | 35,912,157 | 32,383,600 |
| LG4 | 374 | 55.6 | 0.15 | 51 | 19-21 | 8.5 | 28,442,674 | 29,030,946 |
| LG5 | 344 | 60.9 | 0.18 | 50 | 19-22 | 12.0 | 35,745,824 | 33,625,428 |
| LG6 | 337 | 77.8 | 0.23 | 51 | 26-28 | 7.9 | 31,026,386 | 32,399,676 |
| LG7 | 397 | 57.2 | 0.14 | 46 | 8-10 | 12.5 | 39,213,461 | 32,077,157 |
| LG8 | 345 | 62.6 | 0.18 | 48 | 9-11 | 12.5 | 31,485,414 | 30,367,305 |
| Total | 3075 | 531.0 | 0.17 | 407 |  |  | 279,897,610 | 264,224,614 |

## Table S5. Summary of statistics of the transcriptome mapping to the red bayberry genome assembly.

| **Genome** | **Data origin** | **Dataset** | **Number** | **Total length (bp)** | **Covered by assembly** | **with >90% sequence in one scaffold** | | **with >50% sequence in one scaffold** | |
| --- | --- | --- | --- | --- | --- | --- | --- | --- | --- |
|  |  |  |  |  |  | **Number** | **Percent（%）** | **Number** | **Percent（%）** |
| female | Y2012-145 transcripts | All | 61,391 | 85,350,612 | 97.5 | 53,454 | 87.1 | 58,012 | 94.5 |
|  |  | >200bp | 61,391 | 85,350,612 | 97.5 | 53,454 | 87.1 | 58,012 | 94.5 |
|  |  | >500bp | 41,384 | 78,901,607 | 99.1 | 35,238 | 85.2 | 39,322 | 95.0 |
|  |  | >1000bp | 30,117 | 70,701,464 | 99.7 | 25,069 | 83.2 | 28,576 | 94.9 |
|  | EST | All | 38,717 | 28,045,175 | 95.2 | 35,041 | 90.5 | 36,684 | 94.7 |
|  |  | >200bp | 38,717 | 28,045,175 | 95.2 | 35,041 | 90.5 | 36,684 | 94.7 |
|  |  | >500bp | 18,204 | 21,645,809 | 98.9 | 17,042 | 93.6 | 17,919 | 98.4 |
|  |  | >1000bp | 8,982 | 15,064,079 | 99.8 | 8,402 | 93.5 | 8,925 | 99.4 |
| male | H2011-12 transcripts | All | 99,328 | 119,301,033 | 96.6 | 92949.00 | 93.6 | 95388.00 | 96.0 |
|  |  | >200bp | 99,328 | 119,301,033 | 96.6 | 92949.00 | 93.6 | 95388.00 | 96.0 |
|  |  | >500bp | 64,014 | 107,474,544 | 97.9 | 60379.00 | 94.3 | 62241.00 | 97.2 |
|  |  | >1000bp | 41,992 | 91,711,852 | 98.6 | 39643.00 | 94.4 | 41040.00 | 97.7 |

## Table S6. The statement of the categories of BUSCO groups searched in red bayberry genome

| **Classify** | **Number** | **Percent (%)** |
| --- | --- | --- |
| Complete BUSCOs (C) | 1357 | 94.24 |
| Complete and single-copy BUSCOs (S) | 1096 | 76.11 |
| Complete and duplicated BUSCOs (D) | 261 | 18.13 |
| Fragmented BUSCOs (F) | 27 | 1.88 |
| Missing BUSCOs (M) | 56 | 3.89 |

Table S7. Classification of red bayberry repeat sequences (in Excel file).

Table S8. Tissue source for RNA-seq and total amount of available sequence data (in bp).

| **Library** | **Material** | **Tissue** | **Clean data (bp)** |
| --- | --- | --- | --- |
| Root | Y2012-145 roots collected in March 25th, 2015 | root | 7,379,297,100 |
| Stem | Y2012-145, stem collected in May 8th, 2015 | stem | 6,735,065,400 |
| Leaves | Y2012-145, leaves colletcted in April 16th and May 8th, 2015, pooled together | leaves + petioles | 7,595,692,500 |
| Buds | Y2012-145, female floral buds and flower collected in March 25th and April 16th, 2015, pooled together. | buds + flower | 9,242,362,500 |
| small fruit | Y2012-145, small fruits collected in May 8th, 2015. | fruit | 7,287,297,750 |
| Young fruit | Y2012-145, young fruit collected in June 1st, 2015. Three biological replicates | fruit | 29,215,999,500 |
| Break fruit | Y2012-145, break fruit collected in June 11th, 2015. Three biological replicates | fruit | 37,475,109,000 |
| Mature fruit | Y2012-145, mature fruit collected in June 23rd, 2015. Three biological replicates | fruit | 29,505,430,800 |
| Male buds | H2011-12, male floral buds and flower collected in December 11th and 26th, 2015, January 26th, and February 21st, 2016, pooled together. | buds + flower | 6,640,001,400 |
| Male flowers-A | H2011-12, male flower collected in March 3rd and 14th, 2016, pooled together. Three biological replicates | flower | 21,240,515,100 |
| Male flowers-B | H2011-12, male flower collected in March 10th, 2013. | flower | 4,933,902,420 |

## Table S9. General statistics for predicted protein-coding genes for female red bayberry.

| **Annotation methods** | | **Number of genes predicted in *M. rubra*** | **Average length of transcript (bp)** | **Average length of CDS (bp)** | **Average exon per gene** | **Average length of exon (bp)** | **Average length of intron (bp)** |
| --- | --- | --- | --- | --- | --- | --- | --- |
| *De novo* | fgenesh | 28,759 | 3240 | 1144 | 5 | 210 | 470 |
|  | Augustus | 37,302 | 2488 | 1062 | 5 | 209 | 350 |
|  | GlimmerHMM | 44,792 | 1800 | 791 | 3 | 244 | 451 |
| *Homolog* | *O. sativa* | 23,759 | 2679 | 983 | 4 | 247 | 568 |
|  | *A. thaliana* | 32,101 | 2404 | 906 | 4 | 245 | 555 |
|  | *S. lycopersicum* | 36,121 | 2319 | 882 | 4 | 247 | 560 |
|  | *G. max* | 32,231 | 2622 | 944 | 4 | 238 | 567 |
|  | *P. persica* | 31,981 | 2578 | 1024 | 4 | 251 | 504 |
| GLEAN set | | 33,641 | 2945 | 1046 | 4 | 234 | 546 |
| RNA-Seq | | 31,056 | 2649 | 839 | 4 | 220 | 641 |
| Final gene | | 29,414 | 3339 | 1145 | 5 | 236 | 520 |

## Table S10. Functional annotation of predicted genes for female red bayberry.

|  | Number | Percentage (%) |
| --- | --- | --- |
| Total | 29,414 | 100 |
| Annotated | 26,316 | 89.5 |
| GO | 16,328 | 55.5 |
| InterPro | 21,943 | 74.6 |
| KEGG | 15,684 | 53.3 |
| SwissProt | 20,245 | 68.8 |
| TrEMBL | 26,023 | 88.5 |
| Unannotated | 3,098 | 10.5 |

Table S11. Comparison of red bayberry transcription factors with other species in number observed per transcription factor class (in Excel file).

## Table S12. Identification of non-coding RNA genes in the red bayberry genome.

| **Type** | | **Copy** | **Average length (bp)** | **Total length (bp)** | **% of genome** |
| --- | --- | --- | --- | --- | --- |
| miRNA | | 128 | 127 | 16268 | 0.00520 |
| tRNA | | 626 | 75 | 47170 | 0.01509 |
| rRNA | rRNA | 489 | 178 | 87241 | 0.02790 |
|  | 18S | 49 | 720 | 35302 | 0.01129 |
|  | 28S | 72 | 129 | 9256 | 0.00296 |
|  | 5.8S | 16 | 146 | 2331 | 0.00075 |
|  | 5S | 352 | 115 | 40352 | 0.01291 |
| snRNA | snRNA | 205 | 120 | 24631 | 0.00788 |
|  | CD-box | 128 | 101 | 12870 | 0.00412 |
|  | HACA-box | 1 | 124 | 124 | 0.00004 |
|  | splicing | 76 | 153 | 11637 | 0.00372 |

## Table S13. The statistics of gene families among different species.

| **Species** | **Genes number** | **Genes in families** | **Unclustered genes** | **Family number** | **Unique families** | **Average genes per family** |
| --- | --- | --- | --- | --- | --- | --- |
| *Actinidia chinensis* | 39,761 | 30,348 | 9,413 | 14,367 | 1,162 | 2.1 |
| *Arabidopsis thaliana* | 26,829 | 23,419 | 3,410 | 13,025 | 665 | 1.8 |
| *Morella rubra* | 29,414 | 25,741 | 3,673 | 13,707 | 867 | 1.9 |
| *Carica papaya* | 24,035 | 18,645 | 5,390 | 13,000 | 428 | 1.4 |
| *Citrus sinensis* | 41,674 | 38,019 | 3,655 | 13,683 | 757 | 2.8 |
| *Fragaria vesca* | 32,687 | 25,679 | 7,008 | 14,453 | 1,346 | 1.8 |
| *Populus trichocarpa* | 40,787 | 34,550 | 6,237 | 14,956 | 840 | 2.3 |
| *Vitis vinifera* | 27,931 | 22,265 | 5,666 | 14,148 | 578 | 1.6 |
| *Morus notabilis* | 27,085 | 20,612 | 6,473 | 14,281 | 706 | 1.4 |
| *Solanum lycopersicum* | 33,560 | 25,983 | 7,577 | 14,124 | 927 | 1.8 |
| *Theobroma cacao* | 30,124 | 23,541 | 6,583 | 14,763 | 451 | 1.6 |
| *Prunus persica* | 22,701 | 20,637 | 2,064 | 13,306 | 196 | 1.6 |
| *Medicago truncatula* | 50,444 | 32,191 | 18,253 | 12,709 | 3,644 | 2.5 |
| *Betula pendula* | 27,324 | 21,217 | 6,107 | 14,214 | 550 | 1.5 |
| *Juglans regia* | 32,448 | 27,062 | 5,386 | 14,585 | 577 | 1.9 |

Table S14. GO cluster/analysis for genes in *M. rubra* unique families (in Excel file)**.**

Table S15. Plant materials used for BSA sequencing, resequencing and sex specific primer PCR amplification (in Excel file)

Table S16. Plant materials used for resequencing and BSA.

| **Accession** | **Library** | **Average reads Length(bp)** | **Sex** | **Clean base (Gb)** | **Genome coverage (x)** |
| --- | --- | --- | --- | --- | --- |
| Biqi | Illumina PE | 150 | ♀ | 18.8 | 58 |
| Dongkui | Illumina PE | 150 | ♀ | 12.2 | 38 |
| Xiazhihong | Illumina PE | 150 | ♀ | 11.7 | 36 |
| C2013-14 | Illumina PE | 250 | ♂ | 13.9 | 43 |
| Y2010-7 | Illumina PE | 250 | ♂ | 14.0 | 44 |
| Y2015-20 | Illumina PE | 250 | ♂ | 14.9 | 46 |
| BSA-F | Illumina PE | 150 | ♀ | 34.4 | 107 |
| BSA-M | Illumina PE | 150 | ♂ | 41.0 | 127 |

Table S17. The filtered SNP information in BSA and re-sequenced individuals (in Excel file).

Table S18. Female specific-genes and their paralogous on the same chromosome 8 **(**in Excel file**).**

Table S19. Primer sequences information (in Excel file)**.**
